# Quantitative shape analysis with weighted covariance estimates for increased statistical efficiency

**DOI:** 10.1186/1742-9994-10-16

**Published:** 2013-04-02

**Authors:** Hossein Ragheb, Neil A Thacker, Paul A Bromiley, Diethard Tautz, Anja C Schunke

**Affiliations:** 1 Imaging Sciences, Faculty of Medical and Human Sciences, University of Manchester, Manchester, UK; 2 Max-Planck Institute for Evolutionary Biology, , Plön, Germany

## Abstract

**Background:**

The introduction and statistical formalisation of landmark-based methods for analysing biological shape has made a major impact on comparative morphometric analyses. However, a satisfactory solution for including information from 2D/3D shapes represented by ‘semi-landmarks’ alongside well-defined landmarks into the analyses is still missing. Also, there has not been an integration of a statistical treatment of measurement error in the current approaches.

**Results:**

We propose a procedure based upon the description of landmarks with measurement covariance, which extends statistical linear modelling processes to semi-landmarks for further analysis. Our formulation is based upon a self consistent approach to the construction of likelihood-based parameter estimation and includes corrections for parameter bias, induced by the degrees of freedom within the linear model. The method has been implemented and tested on measurements from 2D fly wing, 2D mouse mandible and 3D mouse skull data. We use these data to explore possible advantages and disadvantages over the use of standard Procrustes/PCA analysis via a combination of Monte-Carlo studies and quantitative statistical tests. In the process we show how appropriate weighting provides not only greater stability but also more efficient use of the available landmark data. The set of new landmarks generated in our procedure (‘ghost points’) can then be used in any further downstream statistical analysis.

**Conclusions:**

Our approach provides a consistent way of including different forms of landmarks into an analysis and reduces instabilities due to poorly defined points. Our results suggest that the method has the potential to be utilised for the analysis of 2D/3D data, and in particular, for the inclusion of information from surfaces represented by multiple landmark points.

## Introduction

The introduction of geometric morphometrics has laid the foundations for a quantitative description of shapes and shape differences, thus revolutionising the century old quest for comparing anatomical features of organisms [1]. It is now also increasingly used to link quantitative descriptions of shape with developmental processes and associated genetic factors [2]. This process generally involves the construction of a parametric model based upon exemplar biological shape specimens, and the most popular of these are linear models. These are used to quantify and predict the correlations in shape variation between and within species. The objectives of this paper are to improve the statistical efficiency of analysis techniques used in the genetic interpretation of shape variation (morphometrics) and to broaden the scope of problems which can be tackled with shape analysis tools. In particular we believe that much shape data is not suitable for use in current approaches, and ‘semi-landmarks’ (those poorly localised in one direction and the majority of measurements for smooth 3D shape) cannot be appropriately utilised [3,4].

Over a decade during the 70’s, bio-mathematical and biometrical aspects of biological shape studies were treated separately. This early work was later criticised during the 80’s by Bookstein [5], Goodall [6] and Kendall [7]. Later, Bookstein [8] worked towards converging notations from Goodall, Kendall and himself, for the biometric analysis of landmark data in a bio-mathematically interpretable framework of shape. As a consequence of these efforts, the standard method for analysis of variation in landmark position is generally regarded as ‘Procrustes’. It comprises a least-squares alignment of a set of landmark features to a mean shape, and this is often followed by eigenvector analysis of the linear correlations in variation around that mean. While the technique is now very popular the approach has several limitations with regard to the types of variation with which it can deal. One of these limitations is due to the assumption associated with taking least-squares differences and eigenvector summaries of distributions. Though many regard these as simply definitional, and in particular associated with ‘shape’, any statistical interpretation suggests that data are measures with homogeneous noise. On the other hand, the Mantel test [9,10] has sometimes been used as an alternative to Procrustes distance to compute correlation between distance matrices (usually symmetric). Though many papers have been published in this area, we are aware of no work in this, or any related, area of point distribution modelling that has provided a framework to allow data to be analysed according to a measurement process.

Although landmarks are generally carefully chosen in order to allow accurate measurements of position within the image, problems will occur if ‘semi-landmarks’, measured from smooth curves or surfaces and only accurately localised in one dimension, are input to the analysis. Landmarks with a high degree of variability can act as outliers in the alignment stage, generating correlated compensating shifts and rotations of the other points. As PCA aims to describe the main sources of variation, high levels of such correlated movement will then necessarily contaminate the extraction of eigenvectors [11]. This contamination cannot be considered a generic variation, as it has occurred purely due to the uncertainty in the measurement. This in turn follows from the subjective definition of the landmark leading to the view that problems can be avoided via appropriate definition. The mathematical concept of homology (and mapping) underlies many of the considerations behind much theoretical work that is described with the mathematical formalisms of isomorphism. Because of such restrictions on the definition of landmarks, semi-landmarks were introduced [12] in order to allow inclusion of other points which are not homologous among the specimens. By this we mean that a unique corresponding location can not be defined. Measurement at these locations must be regularised by a constraint, such as bending energy [12,13], in order to recover the information missing due to the nature of local structure.

From a statistical perspective a homology (in this context) must be augmented by distributions indicative of the extent to which a correspondence can be established. The standard way to deal with inappropriate weighting of data in a least-squares fit is to generalise the least-squares cost to a Mahalanobis distance, computed using measurement covariances. By avoiding the requirement of specifying a unique homologous location, this has the advantage of accommodating varying precision in measured data without having to try to re-create missing data. There have been several attempts in the literature to include measurement errors for landmark points. For example, Fitzpatrick et al. [14] worked on the relationship between localisation error and registration error in rigid-body, point-based registration. Chui and Rangarajan [15] proposed a general framework for non-rigid point matching, where outliers are effectively rejected. Rohlf and Slice [16], and Walker [17] investigated how to estimate measurement covariances in forms. However, Richtsmeier et al. [18], Adams et al. [1] and Rohlf [19] all stated that further research was needed in this area. Also, Walker [17] and Lele [20] concluded that generalised Procrustes analysis (GPA) estimators of the variance-covariance matrix are flawed. Despite the fact that some biologists have noticed these problems, they seem to know of no available alternatives and continue to use GPA to estimate covariances [21].

Text books [22] state that using weighted Procrustes does not lead to a Kendall’s shape space. Claiming that “statistical analysis cannot employ parametric models”, they suggested that resampling-based methods must be used instead. Another reason for rejecting the idea of a weighted Procrustes was said to be a “lack of clear criteria for determining appropriate weighting of semi-landmarks”. These criticisms can only really be interpreted once a method for weighting is specified. Goodall [23] suggested a method in which the same covariance was used for all landmarks. By this we mean there was no separate description of the perturbation of individual landmarks. It has been noted that such a matrix is inestimable [24]. Goodall himself acknowledged that “as a model of measurement error this is a drawback, as the direction of greatest variation may vary considerably between landmarks”. Despite this problem, later work [25] generalised this idea to a Bayesian framework. We believe that it makes sense instead to suggest an approach which can support the process of landmark location as measurement, with a covariance describing the localisation of each landmark separately (see [26,27] for example). Specifically, Rohr et al. [26] used covariance matrices in a Mahalanobis distance form for non-isotropic data, where covariances were estimated from image data through landmark localisation, i.e. using grey-value information from local pixels around each landmark for matching an image area/volume structure to another through optimisation of a cost function. The minimal localisation uncertainty for each point were estimated using the Cramer-Rao Bound (CRB). Also, smoothness was included as the second term in their functional and controlled using a regularisation parameter. To our knowledge, they have been the first to provide a relatively comprehensive approach for incorporating anisotropic covariances into image registration using splines. However, here we only deal with pre-defined landmark data and, unlike their method (and our recently published method [28]), do not attempt to extract landmarks and their corresponding covariances from image data. Specifically, in [28], we have applied smoothing to local edge data (where information is) prior to optimisation in order to remove the effects of spatial noise and obtain meaningful CRB estimates. However in our current study, the only input data fed to our method are a number of shapes represented by fixed landmark points. Hence, we do not take into account any information about the local structure surrounding each landmark. This way, the task of covariance estimation may be seen even more challenging. We are aware that in biological studies it is now commonly accepted that for point-based shapes, extra information about the local/global pixels in the image plane/volume (for 2D/3D data) is usually available using modern imaging equipment. However, here our observation is that geometric morphometrics should originally be capable of dealing with the study of 2D/3D forms [18] even for non-biological data or cases where information about the local structure around each landmark is missing or difficult to access or process. It is worth mentioning here that one reason why Procrustes still is popular is that apart from the forms (shapes) represented by landmark points it does not require any further data such as images from which the points have been originated. Hence, even though the datasets we use in our experiments are biological and one could also feed in the image data, in this study we chose to start the process from pre-defined landmarks only. Ideally, covariances extracted from image data (using other methods such as ours [28]) could be fed to our current method and be used, for instance, as initial estimates. This is however a subject for future investigation.

There have been further publications on anisotropic weighting, for instance in [29,30]. Mathematically, these methods are all equivalent to our approach, in that they use a Mahalanobis distance based upon anisotropic distributions of individual points. However, they do not have a well-defined mechanism for the estimation of these distributions. This is a key issue when applying these ideas to shape samples. Our work provides such a mechanism while incorporating corrections for estimation bias [7]. The basic concept can be implemented via a standard technique used in pattern recognition, often referred to as whitening [31]. For instance, in the context of shape analysis, the whitening transform and shape de-correlation were used as a preprocessing step in PCA/ICA analysis [32,33]. However, there is a difference between using whitening methods to model the signal variation of data (as used in these papers) and using the same technique to better construct a likelihood function that accounts for correlation in measured data (as we do here). Recently, the technique has been applied to the within group biological covariances [34], but again not to the process of noise on measurements. Here, we shall investigate possible generalisations of Procrustes along these lines, and the different ways such a measurement covariance may be estimated. As a key issue here is the computability of these covariances, the stability of the resulting analysis is an important question for investigation. The theory presented here can thus be classified in the same category as both Procrustes based shape analysis [35] and active shape models [36]. The main difference, however, being that our model is for a realisable system and self-consistent estimation of the associated model parameters.

There has been an ongoing discussion in the biology literature regarding appropriate ways to deal with non-homologous landmarks (points defined on smooth curves and surfaces) during statistical analysis. For instance, Klingenberg [37] has objected to Polly’s conclusions [38] regarding the benefits of existing homology-free approaches. He believes that these approaches all depend critically on some sense of homology since they are not really free of assumptions about the correspondence of parts. Oxnard and O’Higgins [39] have recommended that it is biology that has to inform morphometrics in planning the landmark configuration (mainly mathematical landmarks, i.e. those computed using geometric constraints based on the neighbouring true landmarks) in relation to the hypothesis available. The approach to dealing with semi-landmarks in the morphometric analysis of shape currently seems to be divided between two alternatives, both of which aim to adjust the position of these landmarks by optimising a specific metric, before constructing a linear model of variation about the mean. These metrics are bending energy (BE) and Procrustes distance (PD) [3]. Arguments for and against these approaches are based upon specific examples in biology. Although evidence has been reported of utility [40], Slice [41] has stated that the application of the BE approach to biomedical and anthropological problems has been minimal. Vignon and Pierre [4], and Prez et al. [42] have shown concern regarding the observation that different methods for handling semi-landmarks could result in different conclusions in a discriminant analysis study. Gomez-Robles et al. [43] have examined the advantages and disadvantages of different novel methods in geometric morphometric analyses including homology-free approaches, landmark-based approaches, and combinations of both techniques.

Comparison between results from shape analysis and genetics is an important research topic in evolutionary biology. For instance, Frederich et al. [44] have attempted to estimate the statistical correlation between morphological, genetic and geographical distances. We offer an alternative shape analysis method that tackles the existing problem in the literature, so that well defined comparisons become statistically valid and informative.

## Methods

Suppose that there are *K* shapes in our data-set and each shape vector **w**_*k*_ contains *N* landmark points, i.e. **w**_*k*_=[*w*_1*x*_,*w*_1*y*_,*w*_2*x*_,*w*_2*y*_,...,*w*_*Nx*_,*w*_*Ny*_]_*k*_ for the case of 2D data. We then apply a scale *s*_*k*_, a rotation *R*_*k*_ and a translation **t**_*k*_ to the original data to get an aligned version of the data called **z**_*k*_, where **z**_*k*_=[*z*_1*x*_,*z*_1*y*_,*z*_2*x*_,*z*_2*y*_,...,*z*_*Nx*_,*z*_*Ny*_]_*k*_ and **z**_*k*_=*s*_*k*_*R*_*k*_(**w**_*k*_−**t**_*k*_).

The mathematical description of the model so far can accommodate any value of scale or orientation for the definition of mean model. We therefore define the orientation of mean shape so that the line between a specified pair of points is horizontal. This also has the benefit that initial estimates of alignment for sample *k* can be set according to the relative positions of these points. We also use the average distance between these same landmarks to rescale the mean shape at each iteration so that scale remains numerically defined.

For 2D data, we assume a different but fixed 2×2 covariance matrix for each landmark derived from the measurement process. These are composed into the matrix *C*. This is a tri-diagonal matrix, the diagonal line of which contains data for individual landmarks. Outside of the 2×2 covariances, the off diagonal elements of *C* are zero, i.e. there are no correlations between landmarks. The use of a fixed data covariance cancels out when taking the weighted mean, to regenerate the conventional formula for the mean;

(1) $$m=\frac{1}{K}\overset{K}{\underset{k=1}{\sum }}z_{k}$$

where **m**=[*m*_1*x*_,*m*_1*y*_,*m*_2*x*_,*m*_2*y*_,...,*m*_*Nx*_,*m*_*Ny*_]. This definition for mean shape has previously been shown to provide unbiased estimates using Monte-Carlo re-sampling studies [19], which is to be expected for a valid likelihood estimate of parameters.

The points **z**_*k*_ do not have uniform independent noise distributions, which is one of the assumptions for the application of PCA. However, this property can be obtained via a whitening transformation. Although transformation of data can be considered as a new space, it can also be interpreted as an affine re-projection. The points obtained by applying a whitening transformation are referred to here as ‘ghost points’. Ghost points are accordingly defined in the original coordinate system and, being scaled projections relative to the shape centroid, are an alternative way to summarise the original measurement relative to the observable structure. This is an important philosophical issue for those who believe that the original co-ordinate system is somehow more meaningful as a description of biological variation than any linear re-projection (see Discussions). The process amplifies the spatial variation in directions which are well measured relative to those which are not so that the resulting locations have isotropic errors (as required). In turn, this allows accurately measured structure to be encoded in the most significant eigenvectors (those with largest eigenvalues) of the linear model. We transform **z**_*k*_ to ghost points **g**_*k*_ using the matrix *W* so that $g_{k}^{T}=W{\left(z_{k}-m\right)}^{T}$.

By applying singular value decomposition to *C*^−1^, i.e. *C*^−1^=*U*^*T*^*VU*, and making it equivalent to *W*^*T*^*IW*, we find that the required whitening matrix is *W*=*V*^1/2^*U*. Application of PCA to **g**_*k*_ follows for construction of the shape covariance, giving the eigenvectors **e**_*j*_ and eigenvalues *μ*_*j*_ for the whitened space of ghost points as those which minimise the unexplained variance for fixed *J*<*N*, where *J* is the number of eigenvectors used in the model. Hence, for any specific shape example *k*, linear factors *λ*_*jk*_ = **e**_*j*_.**g**_*k*_ can be computed to best approximate **z**_*k*_ with the model $z_{k}^{\prime }$;

(2) $$\;F=\overset{K}{\underset{k=1}{\sum }}g_{k}^{T}g_{k}\approx \overset{J}{\underset{j=1}{\sum }}\mu_{j}e_{j}^{T}e_{j},\;z_{k}^{\prime }=m+W^{-1}\overset{J}{\underset{j=1}{\sum }}\lambda_{\mathrm{jk}}e_{j}$$

A genuine likelihood should be based upon the variation of the data around the assumed model. Failure to do this results in residuals which cannot be meaningfully interpreted ^a^. Using this argument, if we wish to align to the mean shape we should use a covariance that is consistent with the distribution around the model. In order to find the best *R*_*k*_,**t**_*k*_,*s*_*k*_ parameters for each *k*, we minimise a Mahalanobis distance which is given by

(3) $$\log(P_{kz^{\prime }})={\left(z_{k}^{\prime }-z_{k}\right)}^{T}C^{-1}(z_{k}^{\prime }-z_{k})$$

This is simply the likelihood estimate for the location of the shape given the linear model and the assumed measurement covariances and can be interpreted directly as a *χ*^2^ statistic. By replacing *C* with *I* and $z_{k}^{\prime }$ with **m** this reduces to the least-squares function for standard Procrustes. We can therefore interpret this as a generalisation of the standard approach. However, we do not wish to generalise further by using for example PPCA (probabilistic principal component analysis) [45], as an additional assumption of a Gaussian distribution over derived variables is generally invalidated in morphometric data sets.

Use of Eq. (3) requires an initial estimate of the model and transformed data **z**_*k*_. By setting the initial estimates of the measurement covariance *C* to an identity matrix, these parameters are given by the Procrustes result. We can therefore use Procrustes to set up the initial transformation estimates. To reach the best possible alignment using our new method (anisotropic *C*), we iteratively re-estimate *R*_*k*_, **t**_*k*_ and *s*_*k*_ using the assumed **e**_*j*_, **m**, *C*^−1^ and *W*^−1^. This gives us a new **z**_*k*_, and so a new **m** and *F* for construction of **e**_*j*_. For fixed covariances, convergence can be monitored via construction of the total likelihood $\log(P)\;=\;\overset{K}{\underset{k=1}{\sum }}\log(P_{k})$. One may use the final estimates of **z**_*k*_ and $z_{k}^{\prime }$ to construct the sample covariance;

(4) $$C^{\prime }=\frac{1}{K}\overset{K}{\underset{k=1}{\sum }}(z_{k}^{\prime }-z_{k}){\left(z_{k}^{\prime }-z_{k}\right)}^{T}$$

For a well defined likelihood method this covariance should be consistent with the assumed distribution *C*. However, the use of free parameters during alignment and model construction introduces biases that must be addressed in an iterative analysis in order to avoid instabilities, which will now be described.

### Covariance correction

When attempting to estimate *C*, the use of free parameters during model fitting reduces the sample covariance obtained from residuals. This mechanism is precisely that identified in [7], whereby the observable variation in any single shape sample is reduced onto a manifold in 2*N*−4 dimensions for a 2D shape defined for *N* points. This has generally been considered as a bias in the overall data distribution rather than being associated specifically with a statistical estimation error (as here). A possible outcome of this is the over weighting of landmarks leading to a runaway convergence on one landmark, during iterative estimation of *C*. However, this bias effect is estimable and therefore correctable, as will be illustrated by Monte-Carlo simulation (below). For a single scale parameter associated with an approximate linear vector **f** we can use error propagation to estimate the expected average reduction Δ in the covariance for each 2×2 landmark component of the matrix arising from errors in parameter estimates Δ _*n*_*C* as

(5) $$\Delta_{n}C=\frac{{f_{n}}^{T}⊗f_{n}}{f^{T}C^{-1}f}\;,\;\mathrm{\Delta C}=\overset{N}{\underset{n=1}{\sum }}\Delta_{n}C$$

Note that the denominator is the change in *χ*^2^ expected due to a unit change in **f**, and **f**_*n*_ = *D*_*n*_**f** is the 2D component of **f** corresponding to landmark *n* (where *D*_*n*_ is an operator which zeros all but those quantities associated with the *n*th landmark and ⊗ is for outer product between two vectors). For an eigenvector **e**_*j*_ defined in the whitened ghost space, this would suggest a total correction of

(6) $$\Delta C_{e_{j}}\;=\;\frac{W^{-1}{e_{j}}^{T}⊗W^{-1}e_{j}}{W^{-1}{e_{j}}^{T}C^{-1}W^{-1}e_{j}}\;=\;W^{-1}{e_{j}}^{T}⊗W^{-1}e_{j}$$

The known structure of the covariance can be enforced by zeroing relevant off-diagonal terms. The parameters of the linear model, including scale, rotation, translation and linear model weightings can also be treated in this way. If Θ _*i*_ represents one of the direction vectors of these parameters (with 2*N* elements for 2D data), it follows that direction vectors corresponding to translation in x and y directions Θ _1_=[1,0,1,0,...] and Θ _2_=[0,1,0,1,...] are orthogonal, i.e. Θ _1_.Θ _2_=0. Similarly, direction vectors Θ _3_=**m**=[*m*_1*x*_,*m*_1*y*_,*m*_2*x*_,*m*_2*y*_,...] and Θ _4_=[−*m*_1*y*_,*m*_1*x*_,−*m*_2*y*_,*m*_2*x*_,...] corresponding to scaling and rotation are orthogonal, and so Θ _3_.Θ _4_=0. Note that **m** is identical to the mean vector defined in Eq. (1).

Strictly, Kendall’s definition of shape explicitly removes aspects of object transformation before model construction. Joint estimation of shape and alignment parameters is potentially unstable as estimated linear shape parameters can correlate with transformation parameters. Here we stabilise this process by removing first order correlations from the data covariance *F* prior to model construction.

Hence, to orthogonalise the model, we modify ghost points as follows.

(7) $$g_{k}^{\prime }=g_{k}-(g_{k}.{Ŷ}_{i}^{T}){Ŷ}_{i}^{T}\;,\;{Ŷ}_{i}^{T}=W{\hat{\Theta }}_{i}^{T}$$

where the unit vector ${\hat{\Theta }}_{i}$ is the normalised form of Θ _*i*_. The new **g**_*k*_ is computed iteratively using each ${\hat{\Theta }}_{i}$ so that any variation about the mean that could have been described by an alignment parameter is removed from the correlation matrix *F* prior to model construction. The corresponding measurement covariance correction term is hence given by

(8) $$\Delta C_{\Theta_{i}}={\left({\hat{\Theta }}_{i}^{T}C^{-1}{\hat{\Theta }}_{i}\right)}^{-1}({\hat{\Theta }}_{i}^{T}\times {\hat{\Theta }}_{i})$$

Therefore, the measurement covariance is estimated using

(9) $$C=C^{\prime }+\overset{J}{\underset{j=1}{\sum }}\Delta C_{e_{j}}+\overset{I=4}{\underset{i=1}{\sum }}\Delta C_{\Theta_{i}}$$

Using the above formula, the contribution to the *χ*^2^ lost by using a scaling parameter associated with each vector **e**_**j**_ and Θ _*i*_ contributes a value of unity to the *χ*^2^ for each additional independent degree of freedom, totalling *J*+4. Our method for covariance correction is therefore consistent with a degree of freedom correction as described in conventional analysis approaches [46]. As a consequence the covariance estimation process can be considered equivalent to the Expectation-Maximisation (EM) algorithm, both in operation and parameter estimates, so that the conventional proof of convergence is applicable [47].

### Extension from 2D to 3D

Here we outline the mechanism we use to extend 2D shape rotation analysis, and the extraction of corrected anisotropic measurement covariances, to 3D. The methods are demonstrated in the analysis of 3D mouse skull data, both as a test of the theory/software implementation and as an illustration of use for the identification of outlier landmarks.

The extension to 3D data is mainly involved with the mechanism of representing and estimating 3D shape rotations. We define a fixed orientation co-ordinate system from a set of 3D data-points based upon a selection of three landmark points. We then represent a rotation matrix in terms of three separate rotations about the co-ordinate axes. Finally we compute the linear vectors which approximate the first order shifts seen in the 3D points due to these rotations. These are then used in the linearised approximation for sample covariance correction, as described earlier. These extensions are enough to support a quantitative analysis of 3D landmark data, for the estimation of landmark accuracy and identification of outlier data. The mathematical model used is described in detail here and in Appendix A. We provide quantitative tests in the Results and discussion section which demonstrate the numerical stability of the algorithms using Monte-Carlo data.

### Rotation matrix

Our first task is to define a co-ordinate system for a 3D data-set, from which we can define certain basic properties of orientation for the mean shape, and so that individual data samples can be approximately oriented prior to optimisation during linear model construction. In the 2D case this is done by defining the line between two landmark points in the mean model as horizontal. In 3D, in order to stay consistent with the 2D, we define 2 points to establish a horizontal, and then a third to define the vertical relative to the first two.

**Figure 1:**
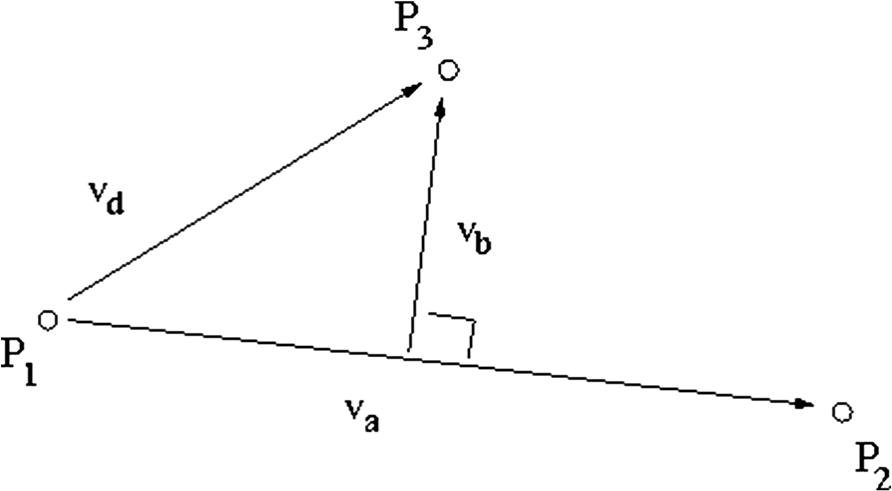
The geometry shows how to define a base-plane in 3D (consistent with the base-line in 2D) using three landmark points.

Given a 3D shape, we take three points *P*_1_, *P*_2_, *P*_3_, with relatively large distance from each other (Figure 1) to define the orientation plane for the shape. The rotation matrix *R*^*T*^ is hence found based on basic vector calculations (see Appendix A).

### Roll, pitch and yaw angles

Given the rotation matrix that brings a data set into alignment with the preferred co-ordinate system it is possible to represent the rotation as a sequence of rotations about three orthogonal axes. According to basic 3D rotation formulas, and using *α*, *β*, and *γ* as yaw, pitch, and roll respectively, the 3D rotation matrix is defined as three consecutive rotations around the z, y, and x coordinate axes.

(10) $$R_{\mathrm{xyz}}=R_{x}(\gamma )R_{y}(\beta )R_{z}(\alpha )$$

By making the rotation matrix *R*^*T*^ equivalent to *R*_*xyz*_, we find the yaw, pitch, and roll angles (see Appendix A). Thus we can convert easily between the rotation matrix and rotation parameters.

### Orientation adjustments

We initialise the rotation angles, by computing the *R*^*T*^ matrix for every original shape in the data set based upon the three identified landmark points and extracting the corresponding *α*, *β*, and *γ* angles. These are then further adjusted during iterative alignment via optimisation of the anisotropic measurement-based Mahalanobis distance. We perform orientation adjustment on the mean shape following every iteration over the set of shape samples. In this case the set of yaw, pitch, and roll angles corresponding to the mean shape are subtracted from the corresponding rotation angles for each shape sample, so that the computed mean shape complies with the three-point orientation constraint.

### Direction vectors

In order to correct the covariances due to alignment parameters in 3D, we need the approximate linear direction vectors corresponding to translation, rotation and scale. Computing these for translation and scale is straightforward. If **m**=[*m*_1*x*_,*m*_1*y*_,*m*_1*z*_,*m*_2*x*_,*m*_2*y*_,*m*_2*z*_,...] is the vector corresponding to the 3D mean shape (with 3*N* elements), then the direction vectors due to translation in x, y and z directions are simply given by Θ _1_=[1,0,0,1,0,0,...], Θ _2_=[0,1,0,0,1,0,...] and Θ _3_=[0,0,1,0,0,1,...]. Also, the direction vector due to scaling is Θ _4_=**m**.

For rotation, we compute the direction vector corresponding to each individual rotations *R*_*z*_, *R*_*y*_ and *R*_*x*_. For the mean shape **m** rotated by the yaw angle *α* around the z axis, we have **m**^*′*^=*R*_*z*_(*α*)**m**. As *α* becomes very small, the tangential direction of movement in landmark point *n* due to this rotation is

(11) $$u_{\alpha \approx 0}=[-m_{\mathrm{ny}},m_{\mathrm{nx}},0]$$

By applying the same method, one can find the direction vectors due to rotation by the pitch angle *β* around the y axis and by the roll angle *γ* around the x axis (see Appendix A). Hence we have

(12) $$\begin{array}{lcrr} u_{\beta \approx 0}= & \left[m_{\mathrm{nz}},0,-m_{\mathrm{nx}}\right] & & \end{array}$$

(13) $$\begin{array}{lcrr} u_{\gamma \approx 0}= & \left[0,-m_{\mathrm{nz}},m_{\mathrm{ny}}\right] & & \end{array}$$

It follows that Θ _5_=[−*m*_1*y*_,*m*_1*x*_,0,−*m*_2*y*_,*m*_2*x*_,0,...], Θ _6_=[*m*_1*z*_,0,−*m*_1*x*_,*m*_2*z*_,0,−*m*_2*x*_,...] and Θ _7_=[0,−*m*_1*z*_,*m*_1*y*_,0,−*m*_2*z*_,*m*_2*y*_,...]. The set of vectors Θ _1_, Θ _2_ and Θ _3_ on one hand, and the set of vectors Θ _5_, Θ _6_ and Θ _7_ on the other hand are mutually orthogonal and orthogonal to the vector Θ _4_ due to scaling. These direction vectors now constitute the linearised parameterisations needed for corrections to the sample covariance (where *I*=7 in Eq. 9).

### Procedures

Here, in Table 1, we provide the step-by-step procedure for our new shape analysis method that involves linear model construction, data alignment and anisotropic covariance estimation and correction. Note that there is an arbitrary order for the application of the transformation parameters which remains consistent throughout the whole process. In fact, whatever this order, the net effect of the covariance correction (Eqs. 7-9) is to subtract the same total linear subspace.

**Table 1:** The algorithmic procedure for our new method

**Step**	**Process**
1	Initialise each translation parameter **t**_*k*_ using the mean of landmarks in each corresponding shape ( *k*=1,2,...,*K*).
2	Initialise each rotation parameter *R*_*k*_ based on the orientation of each shape relative to the 2-point baseline in 2D or 3-point reference plane in 3D (Figure 1).
3	Initialise scale parameters *s*_*k*_ as unity, i.e. original scales.
4	Initialise measurement covariance matrix as identity matrix.
5	Compute initial transformed shapes **z**_*k*_.
6	Compute the initial mean shape **m** (and adjust transformation parameters so that the mean orientation is roughly aligned with the reference baseline/plane).
7	Compute current transformed shapes **z**_*k*_.
8	Compute the current mean shape **m** (Eq. 1).
9	Compute the whitening matrix *W*.
10	Compute current ghost points **g**_*k*_.
11	Construct current models ${\text{z}}_{k}^{\prime }$ based on PCA and the number of eigenvectors **e**_*j*_ chosen *J* (Eq. 2).
12	Minimise the Mahalanobis distance corresponding to every shape **z**_*k*_ (Eq. 3) using simplex optimisation (where **e**_*j*_ and *W* are fixed while **t**_*k*_, *R*_*k*_ and *s*_*k*_, and so, **z**_*k*_, **m**, **g**_*k*_ and ${\text{z}}_{k}^{\prime }$ are varied).
13	Update current estimates of **t**_*k*_, *R*_*k*_ and *s*_*k*_ based on the outcome of the optimisation, and then update current estimates of **z**_*k*_, **m**, **g**_*k*_ and ${\text{z}}_{k}^{\prime }$.
14	Compute current estimate of the sample covariance matrix *C*^*′*^ (Eq. 4).
15	Compute covariance correction term $\Delta C_{{\text{e}}_{j}}$ due to degrees of freedom in the model (Eqs 5-6) for every eigenvector used **e**_*j*_ ( *J*=1,2,...,*J*).
16	Skip this step for the first iteration (as it requires an estimate of *C*); compute covariance correction term $\Delta C_{\Theta_{i}}$ due to parameter orthogonalisation (Eqs. 7-8) for every direction vector Θ _*i*_ corresponding to transformation parameters, *i*=1,2,...,*I* (where *I*=4 in 2D and *I*=7 in 3D).
17	Compute current estimate of the measurement covariance matrix *C* (Eq. 9).
18	Repeat steps 7 to 17 until convergence (typically ≈10 iterations).

### Model selection

A method is needed to select appropriate linear model order based upon the outputs from our analysis. If the linear model is valid then estimated measurement covariances will combine two processes of statistical fluctuation. The first of these will be measurement precision *σ*_*r*_ (our ability to define homologous points reliably), and the second will be due to random (unmodellable) biological variation *σ*_*b*_. So that the observed statistical variation seen in a given direction *v* for any landmark *σ*_*v*_ is

(14) $$\sigma_{v}^{2}\;=\;\sigma_{r}^{2}\;+\;\sigma_{b}^{2}$$

Unfortunately we cannot know the expected value of *σ*_*b*_ in advance. However, the first of these terms can be estimated via reproducibility experiments and compared to the measured directional covariances, using the observation that *σ*_*v*_≥*σ*_*r*_. Thus if we observe individual estimates of measurement covariance which begin to surpass the limiting accuracy known to be set by reproducibility tests, then the model must be over-fitting the data and therefore has too many parameters. We check that for a given model order this inequality is satisfied within statistical limits by considering the principle axes of each landmark measurement distribution. We use a 1% confidence level to set the hypothesis test for over-fitting. This test is expected to be most reliable for the largest variances.

### Monte-Carlo tests and outlier identification

As our method is based on likelihood, we require that the assumed distribution matches the corrected covariance. The standard way to validate this is through generating Monte-Carlo (MC) data using the known distributions. In what follows we experiment with MC data and display a number of informative scatter plots for two forms of test; Test A: When applying our method to the MC data, the mean shape, eigenvectors and measurement covariances used are identical to the ones used when generating the simulated data. Test B: All parameters are estimated using the MC data in order to compare the measurement covariances estimated using the simulated data with those expected, i.e. the ones assumed when generating the MC data.

For Test A the covariances estimated using our method are expected to be within statistical sampling limits of the ones used when generating the MC data. Failure to do so is taken as an indication of a problem with the data sample (i.e. outliers). Outliers can be identified at early stages of analysis as those points which have the largest normalised residual errors.

We use 2.8 standard deviations of the error on the sample variance (or being allowed to have 1% of data falling outside the limits), where the error on the standard deviation *σ* is $\sigma /\sqrt{2(K-1)}$ with *K* being the number of samples [48]. Additional variance is expected for Test B (beyond that seen in Test A), where the linear model must also be estimated. Therefore, having excluded the possibility of outliers using Test A, we can interpret variations beyond the statistical limits as due to instability in linear model construction (specifically the mean and eigenvectors).

#### ***χ***^***2***^ test

A test is needed to confirm the equivalence of measurement covariances computed during repeatability experiments, in order to confirm that our methods generate estimates which are consistent. This can also be done by splitting the data into two separate groups if there are a sufficient number of samples. We perform a modified *χ*^2^ test based upon the construction of corrected covariances on one data set and then used for the calculation of *χ*^2^ for the second set. For large numbers of samples ( *K*>30) the resulting statistic when applied to each 2D landmark is expected to be approximately Gaussian with mean 2*K* and variance 4*K*. We set the statistical test for significant difference on the basis of an allowable range of *χ*^2^/*DoF* corresponding to ±2.8 *S*.*D*., i.e. [0.8, 1.2] for 200 samples. The corresponding plot would confirm the stability of the method if 99% of the *χ*^2^/*DoF* values fall inside the range expected.

#### Fisher information

Fisher information (FI) is a concept for quantifying the constraint on an estimated value associated with data. It has the useful property that the amount of estimated information is linear in the quantity of data. It is generally defined according to the second derivative of a log-likelihood function, but from the association of this function and the CRB we can also observe that, for good model fits, it is proportional to the inverse variance. An empirical estimate of the FI contained in data, and associated with a particular model, can therefore be obtained from the residual distributions following parameter estimation.

We use this idea here to summarise the amount of information that has been extracted from data for a specific analysis. As this quantity scales linearly with the quantity of data it allows us to make comparative statements regarding the statistical efficiency associated with the estimation process. For example, if the FI is seen to double on the same dataset when applying an alternative analysis then this is statistically equivalent to having four times as much data to begin with. A poor analysis method might need a lot more data to reach the same level of statistical equivalence in a hypothesis test than a good method.

### Ethics

The animal datasets used in this paper have been approved according to German ethical standards. They were registered under number V312-72241.123-34 (97-8/07) and approved by the ethics commission of the Ministerium für Landwirtschaft, Umwelt und ländliche Räume on 27.12.2007.

## Results and discussion

We have used example datasets to investigate the stability of covariance weighted shape analysis and to compare quantitative performance figures to the standard approach using Procrustes. We have selected several datasets in order to demonstrate behaviour with different quantities of data, data dimensionality (i.e. 2D and 3D) and model order.

As standard methods, even those including landmark weighting, are not conventionally used in a way that would support estimation of landmark variability we have made some assumptions regarding what would be the most straightforward approach. As mentioned earlier, in this paper we are interested in analysing point-based shape datasets without seeking to obtain extra knowledge about local structures surrounding each landmark. Hence, in conjunction with our method, we have not used methods that estimate localisation errors from the original image data such as those described in [26,28]. For Procrustes we use the residuals from the fitted models to make an estimate of landmark measurement error (although this is widely concluded in the literature not to work [22]). For methods that would support anisotropic weighting, we use a variation of our own method (incorporating iterative re-weighted alignment) to estimate the resulting residuals during iterative analysis. The difference between this and our preferred method is the lack of correction for degree of freedom biases, we therefore refer to this as the “uncorrected” method.

### Data

**Figure 2:**
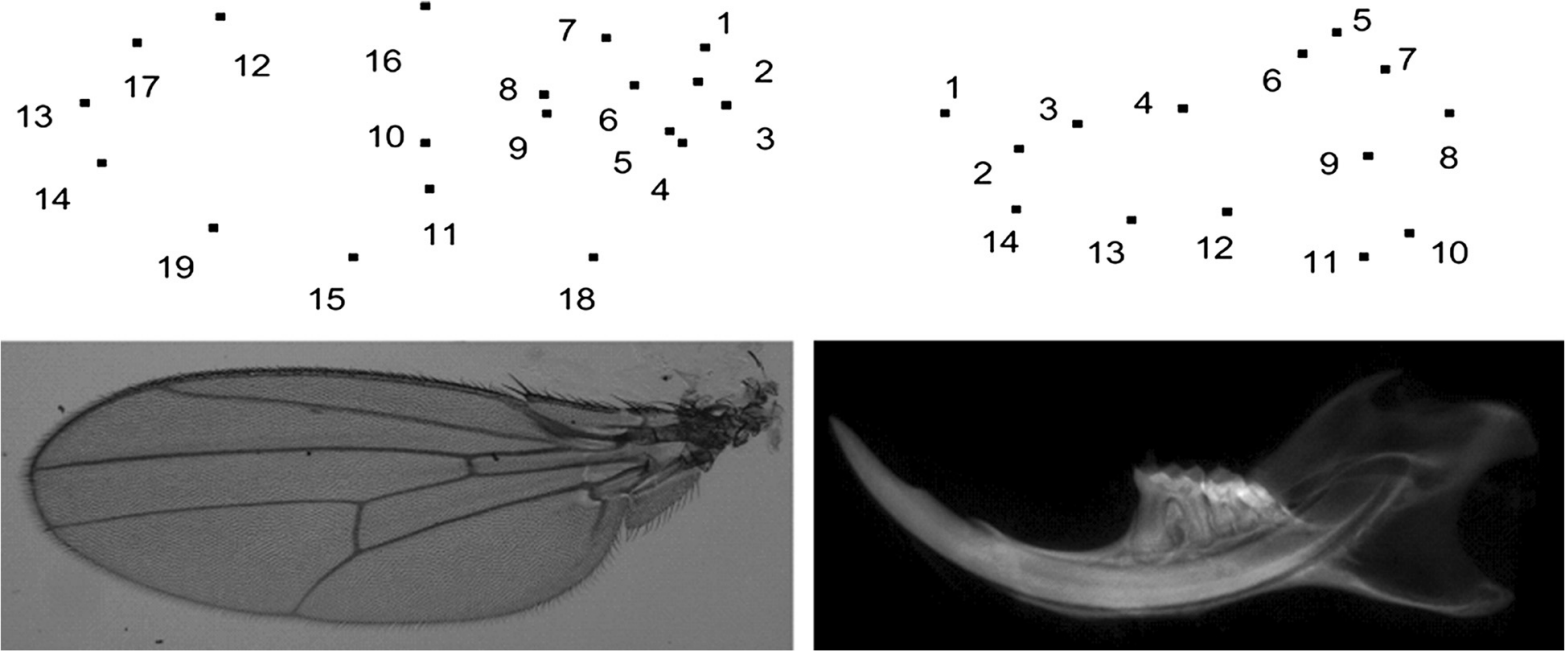
Typical landmarks corresponding to sample images of fly wings (left) and mouse mandibles (right); for the fly wing data, landmarks 1-15 correspond to the original data sets FL1, FL2, FR1 and FR2, while landmarks 16-19 were added later (to FL1) in order to experiment with semi-landmarks (P-FL1).

We experiment with two 2D data sets of manual mark-ups (Figure 2). The first data set, called MM1, corresponds to mouse mandible micro-CT images and consists of 337 samples with 14 landmarks per sample. We also have a repeat data set, called MM2, for which same mandible images have been used to mark-up the points.

Next, we use some fly wing data in order to test the performance of our method on semi-landmarks and also to test the statistical stability of our method. There are four original data sets available from left and right wings(L and R) of 200 female flies, called FL1, FL2, FR1 and FR2 [49]. Two images of each wing were taken from slightly different viewing positions (1 and 2), and used for marking-up in order to perform reproducibility tests [49]. Each of these four data sets has 200 samples with 15 landmarks per sample. Further, as we had access to the fly wing images, we have added four semi-landmarks to each sample of the original data set FL1. Once finished, we removed 5% outliers and stored 189 samples with 19 landmarks per sample. This resulting data set, which is called P-FL1, plays an important role in our experiments with semi-landmarks. In order to be able to test the repeatability with these added semi-landmarks, we have repeated the marking-up process only for the four new landmark points and using a subset of the left fly wing images.

**Figure 3:**
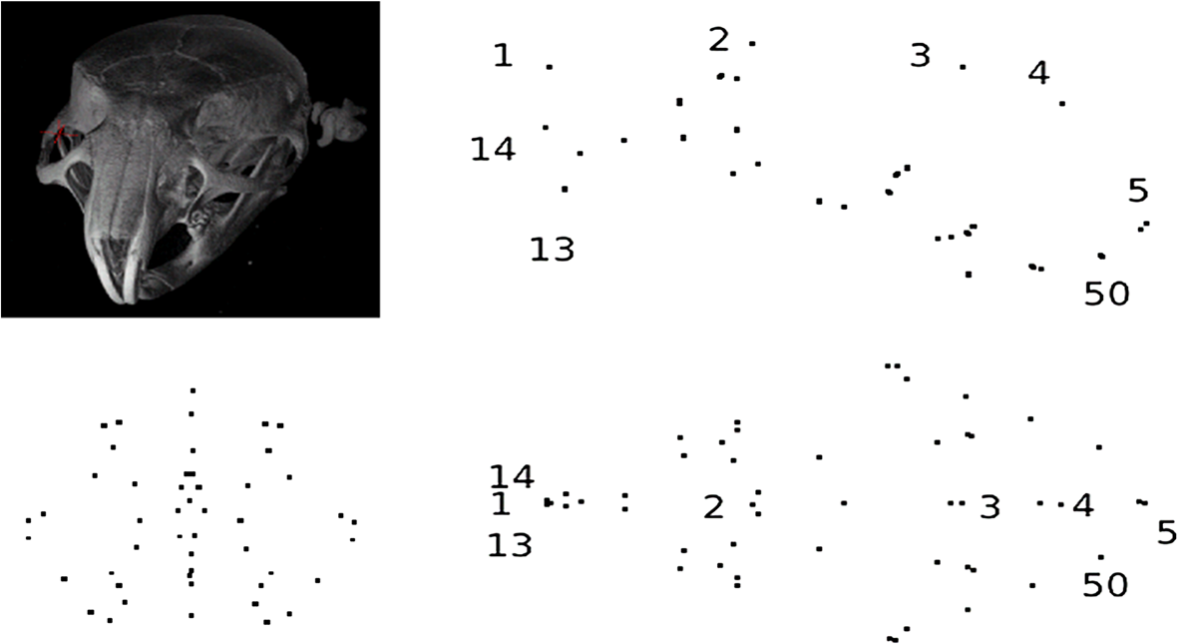
Typical landmarks for a sample volume (top-left) from the 3D mouse skull (MS) data when projected on the xy (top-right), zy (bottom-left) and xz (bottom-right) planes (the 50 points are too close to display full numbering).

We also experiment with the mouse skull (MS) 3D data of semi-automatic mark-ups (Figure 3) produced based on training examples and the corresponding micro-CT images. This makes a typical 3D data set of interest in evolutionary biology research. We have used our automatic tool to localise landmarks on these mouse skulls based on few given manual mark-up examples [50,51]. This is based on landmark localisation technique recently described in [28] (where more details may be found). The automatic tool also identifies outliers for manual correction and so we do not expect any outlier in this data set. The mark-up data set obtained this way (MS) consists of 42 samples with 50 landmarks per sample. Further, there are two sub-sets of repeat data based on manual mark-ups (on the mouse skulls) each consisting of 12 samples to be used in repeatability tests.

### Model selection

**Figure 4:**
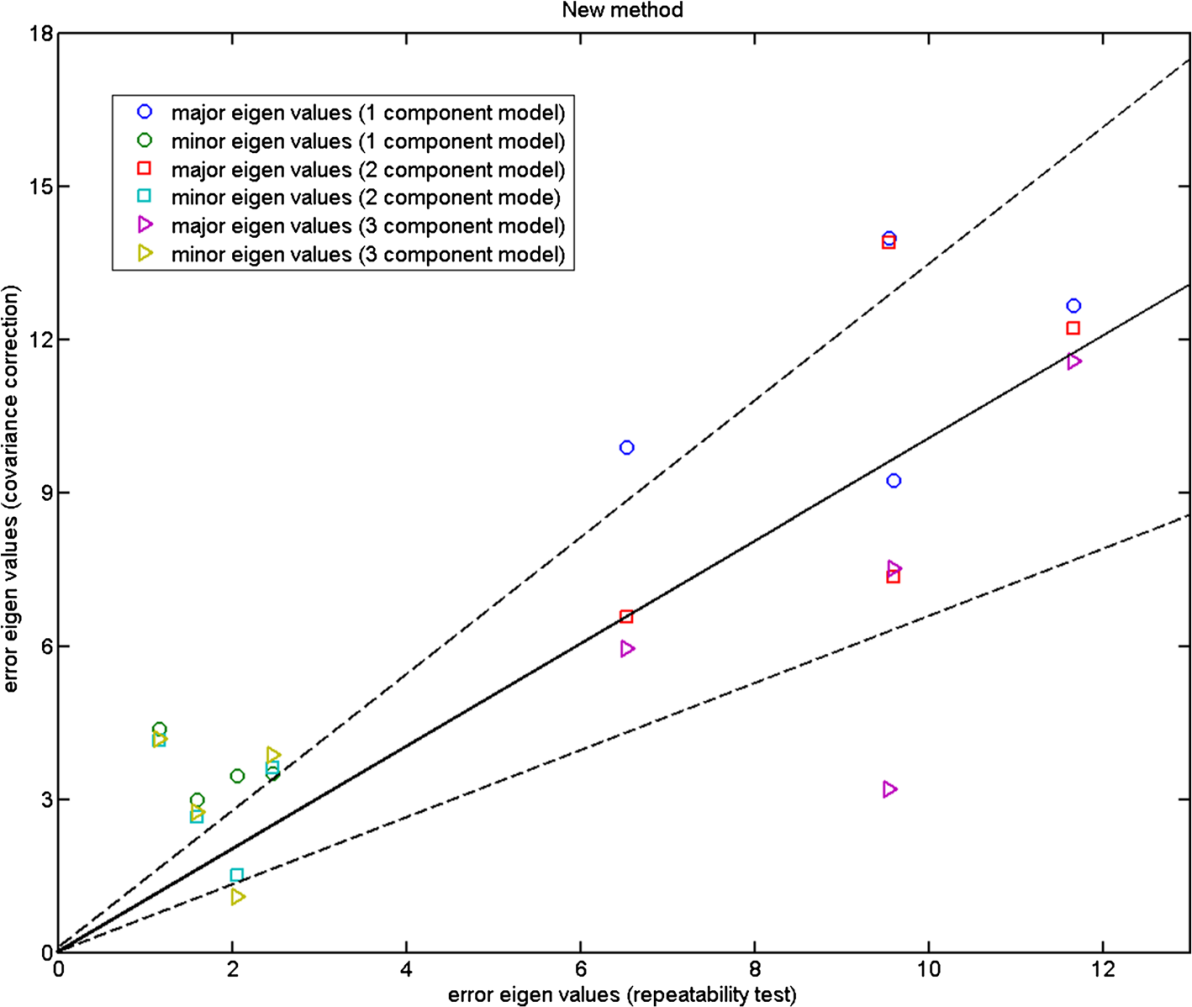
**Fly wing data (P-FL1): major eigenvalues of the error using our 1, 2 and 3 component models against those computed using a repeatability test on four new semi-landmarks placed manually on a subset of data; the 2 component model gives closest agreement to the expected localisation values; the two dashed lines show the*±2.8σ* range.**

**Figure 5:**
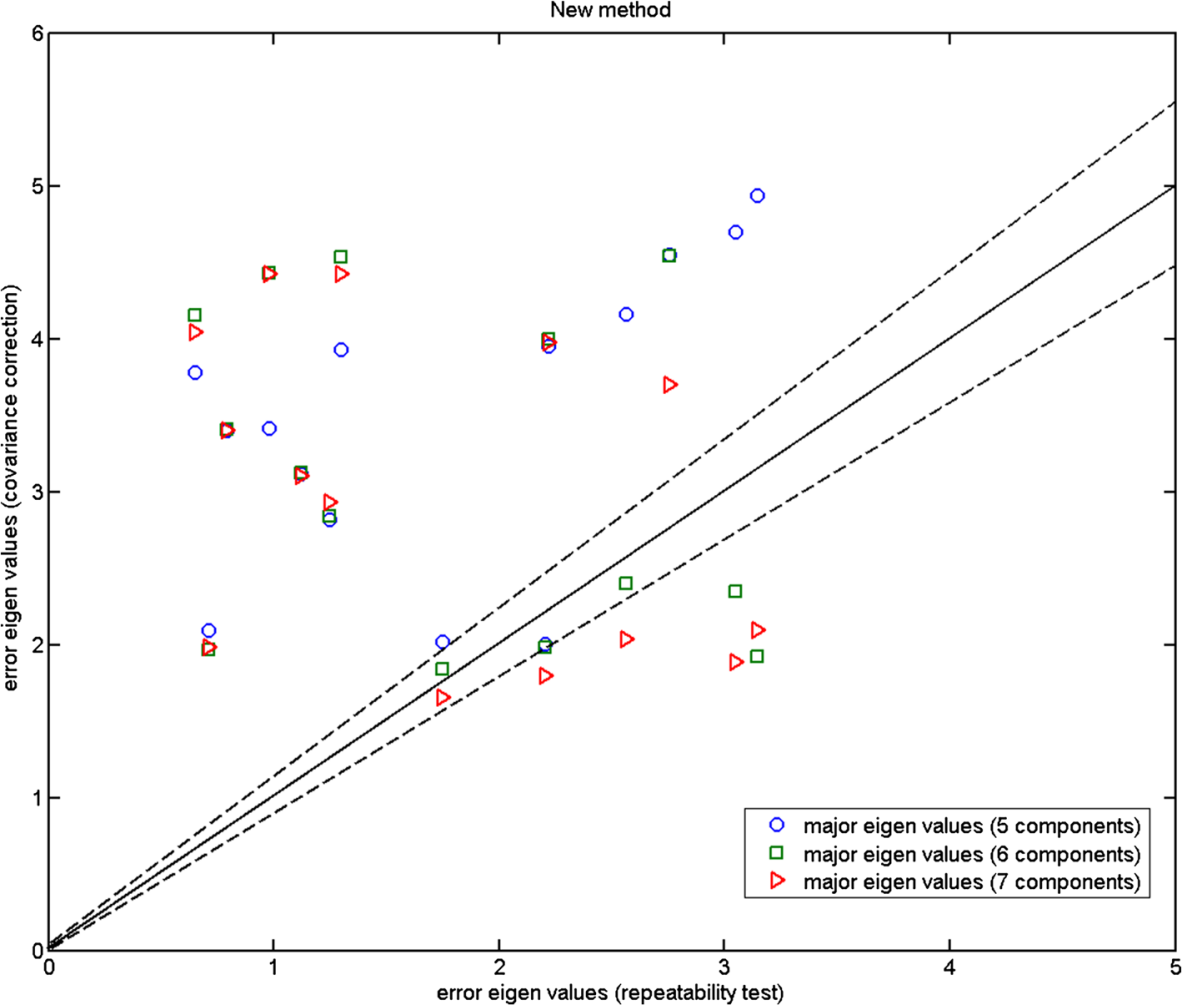
**Mouse mandible data: major eigenvalues of the error estimated using our 5, 6 and 7 component models on MM1 data against those computed using the corresponding repeatability test (MM1 and MM2); the two dashed lines show the*±2.8σ* range.**

**Figure 6:**
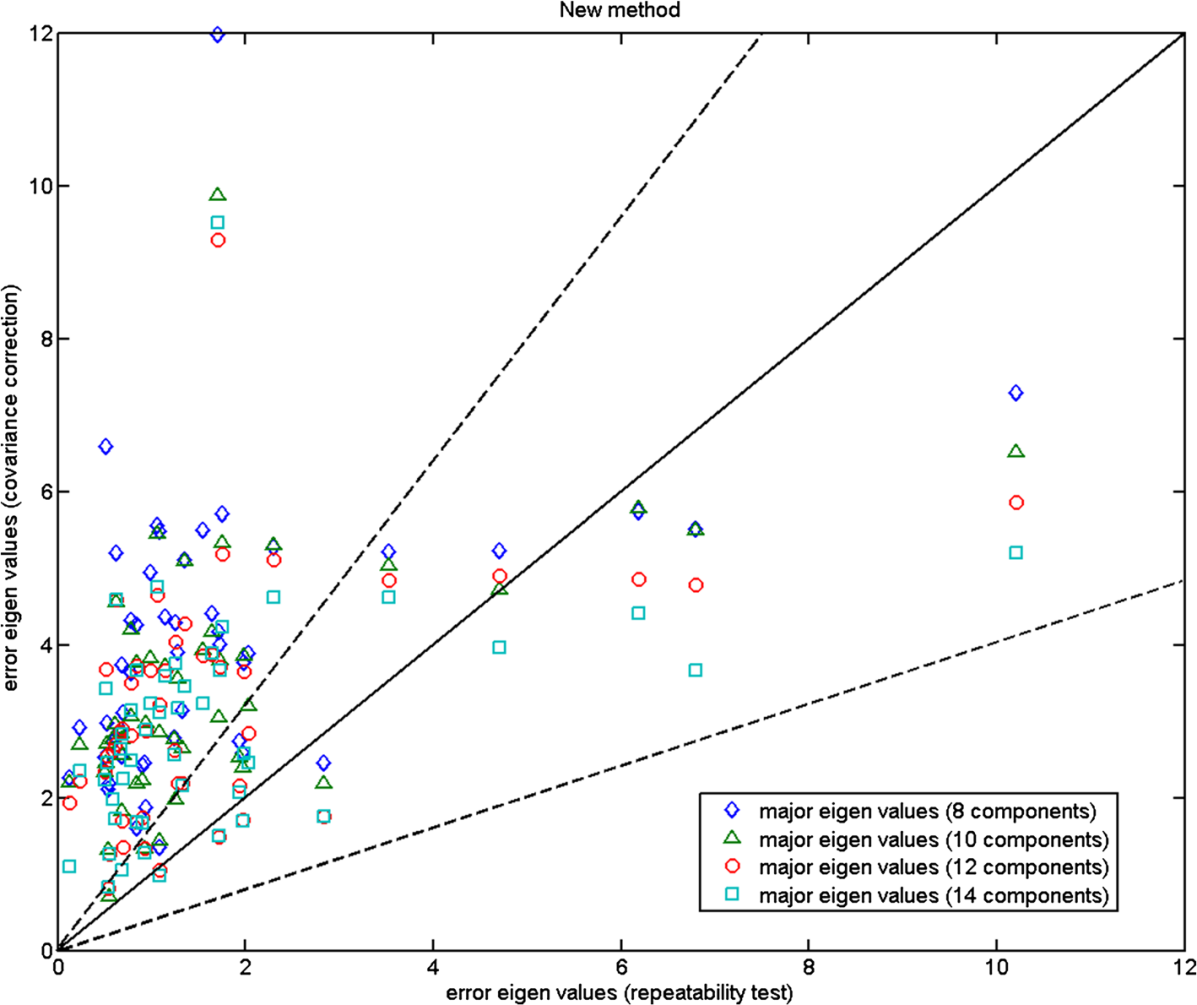
**Mouse skull data: major eigenvalues of the error estimated using our 8, 10, 12 and 14 component models for the 3D MS data against those computed using the corresponding repeatability test; the two dashed lines show the*±2.8σ* range.**

In Figures 4, 5 and 6, we plot the eigenvalues corresponding to the errors estimated against those computed from the repeat data. These are the magnitudes of the errors in the direction of major eigenvectors. It can be seen that while for the fly wing data the errors are comparable (Figure 4), for the mouse mandibles there are several landmarks for which the error estimates are much larger than expected (Figure 5). We cannot argue for an increased model order as this then reduces other values to well below the observed repeatability (over fitting). As the additional variance seen is due to the inability of the model to predict correlations in the data, our conclusion must be that either this data is not well described by a linear model, or the repeatability estimate systematically underestimates the true accuracy with which points can be meaningfully located. This can happen if local image features (which are themselves not well biologically related to the main structures, such as the brightest pixel) are used to identify locations. The plot for the more complex mouse skull data (Figure 6) suggests that 14 components is about the number needed by the linear model. Hence, when experimenting with the 3D MS data we use 14 model components, while using 6 components with the 2D MM data and 2-3 components with the 2D FL and P-FL data. The 1% allowable range is set in accordance with 12 repeat data samples.

### Monte-Carlo tests

**Figure 7:**
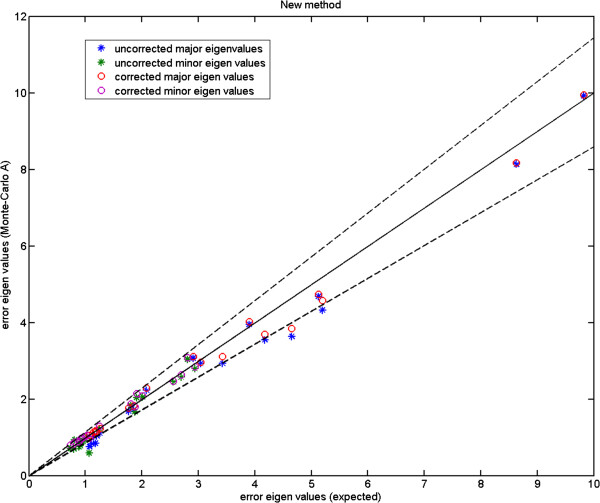
**Fly wing data (P-FL1): error eigenvalues estimated using the Monte-Carlo data (where mean shape, eigenvectors, and measurement covariances are identical to the model which generated the simulated data) against the expected ones (Test A); using 2 model components; there is only marginal evidence of estimation bias before correction;the two dashed lines show the*±2.8σ* range.**

**Figure 8:**
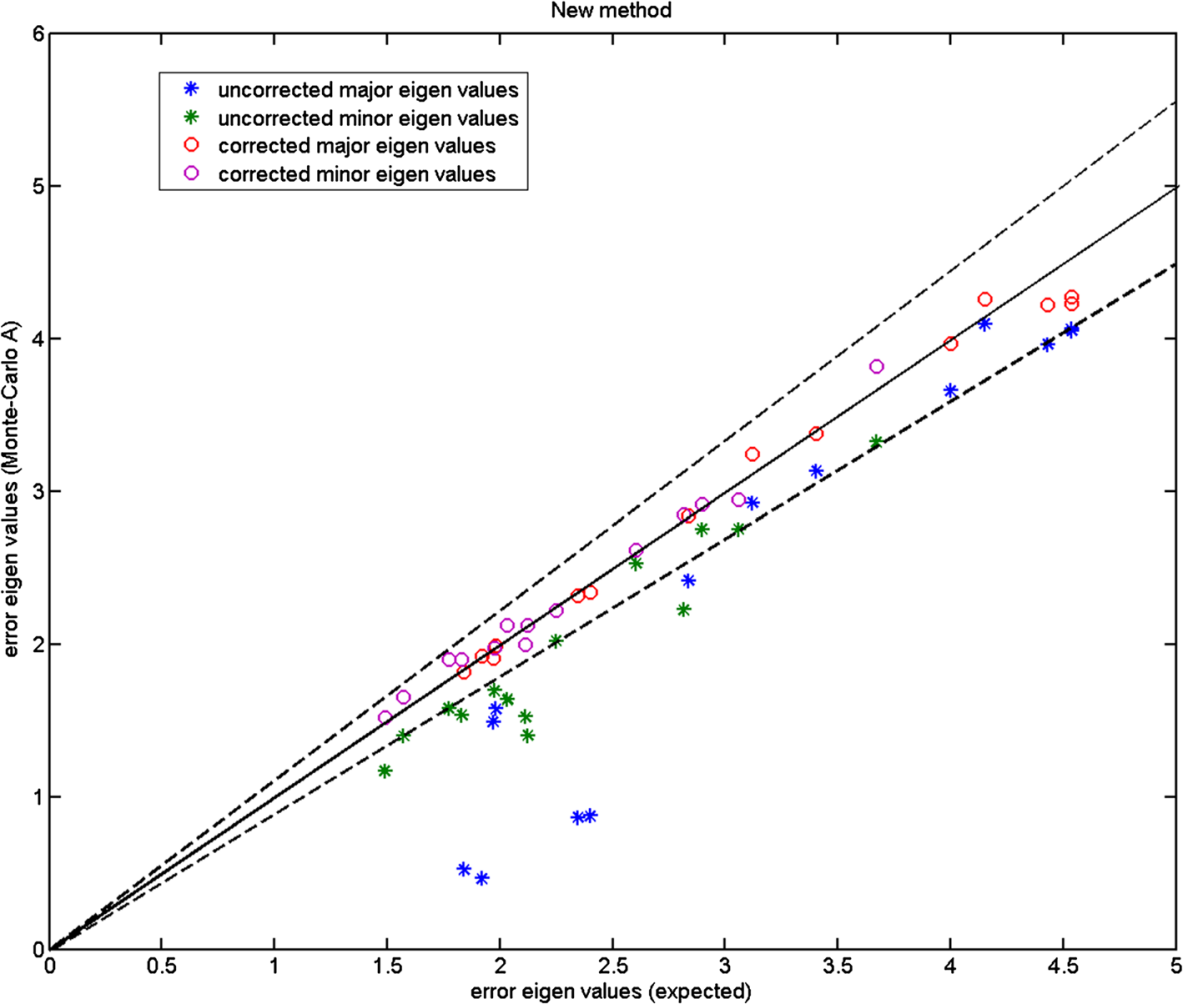
**Mouse mandible data (MM1): error eigenvalues estimated using the Monte-Carlo data (where mean shape, eigenvectors, and measurement covariances are identical to the 6-component model which generated the simulated data) against the expected ones (Test A); for this number of parameters there is now evidence of a systematic underestimate of covariance (prior to correction); the two dashed lines show the*±2.8σ* range.**

**Figure 9:**
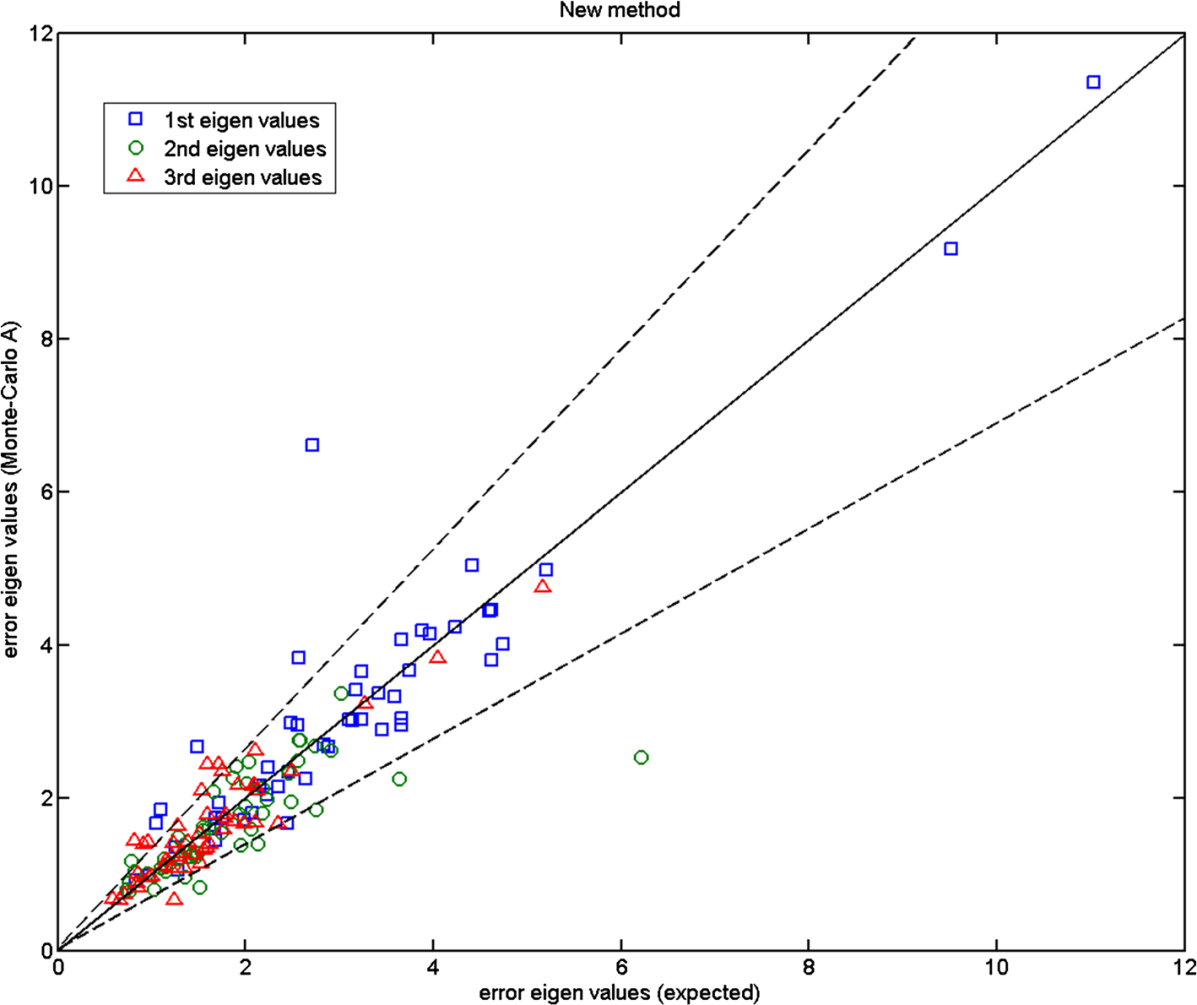
**Mouse skull data (MS): error eigenvalues estimated using the Monte-Carlo data (where mean shape, eigenvectors, and measurement covariances are identical to the 14-component model which generated the simulated data) against the expected ones (Test A); the two dashed lines show the*±2.8σ* range.**

**Figure 10:**
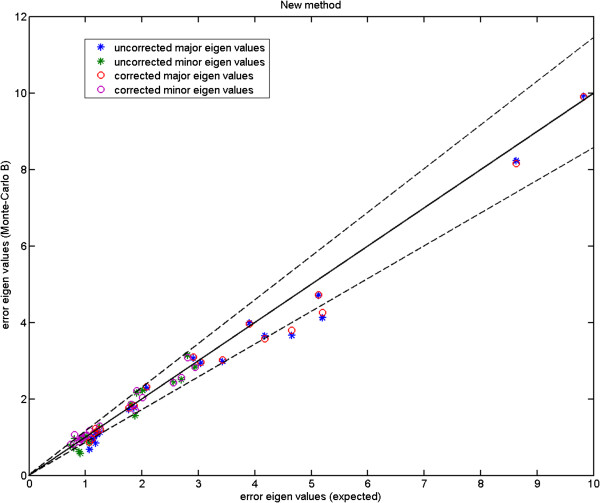
**Fly wing data (P-FL1): error eigenvalues estimated using the Monte-Carlo data against the expected ones (estimated using the original data) which were used when generating the simulated data; independent models (Test B); using 2 model components; the two dashed lines show the*±2.8σ* range.**

**Figure 11:**
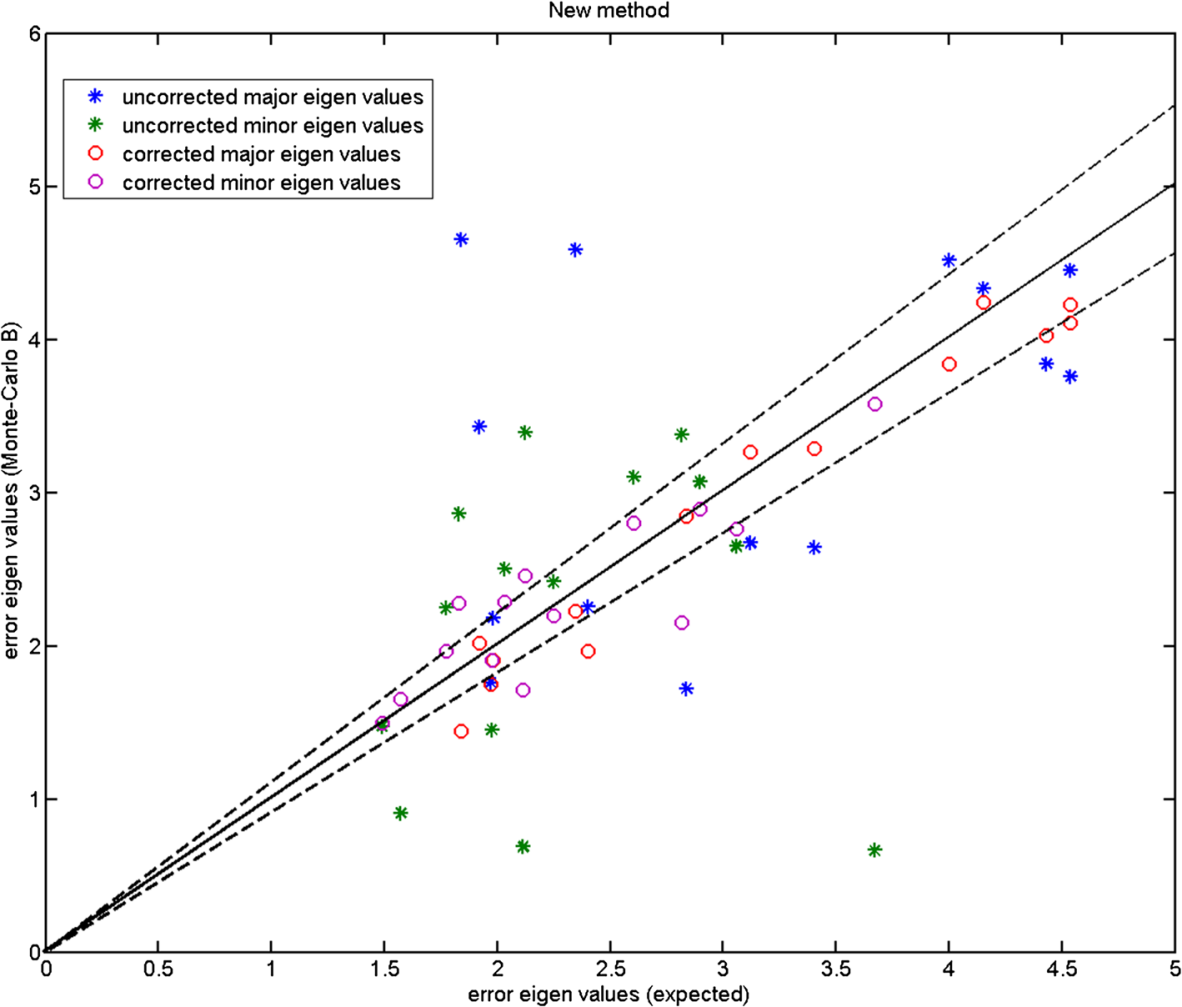
**Mouse mandible data (MM1): error eigenvalues estimated using the Monte-Carlo data against the expected ones (estimated using the original data) which were used when generating the simulated data; independent 6-component models (Test B); for this number of linear model components there is considerable error in the uncorrected estimates; the two dashed lines show the*±2.8σ* range.**

**Figure 12:**
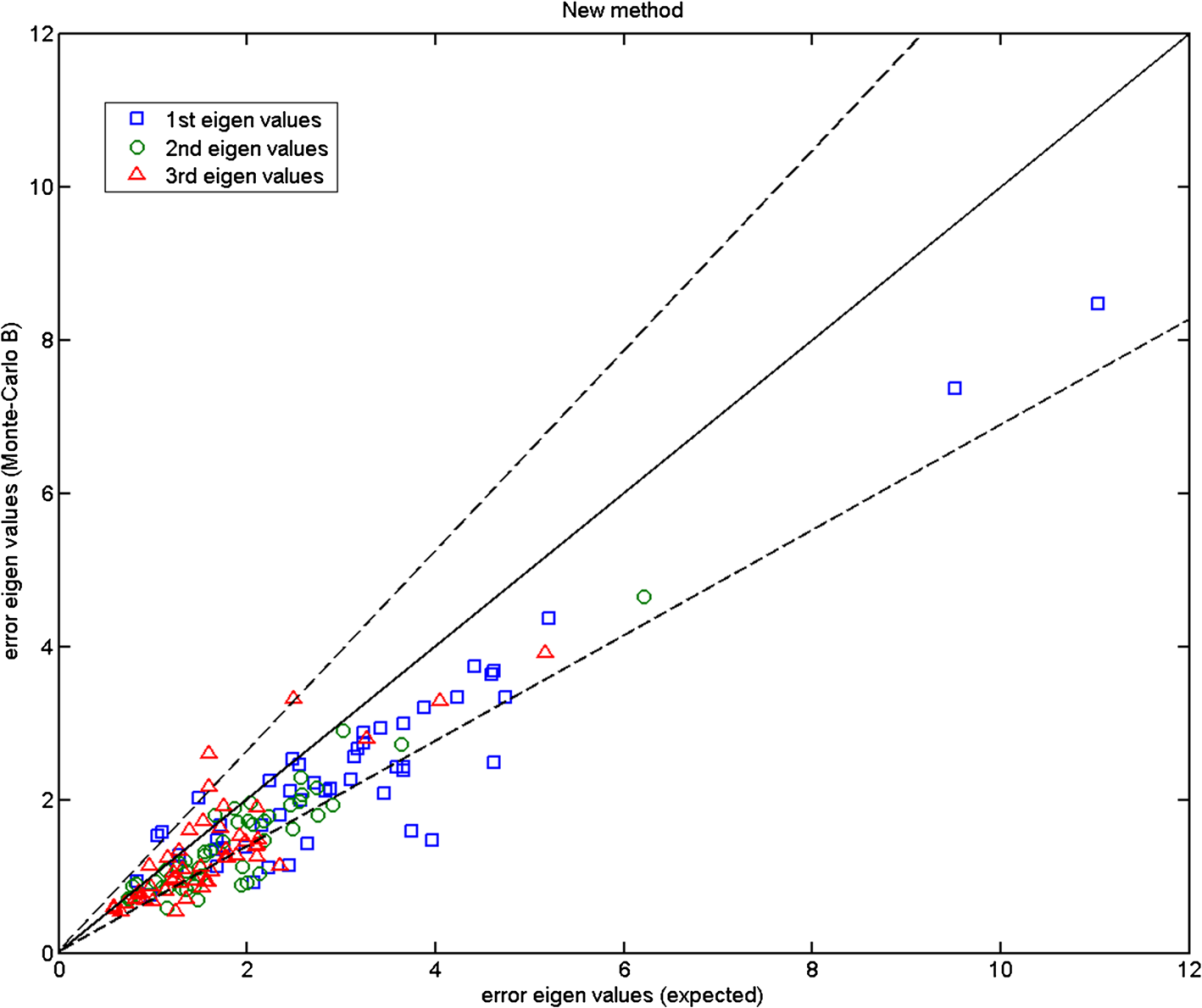
**Mouse skull data (MS): error eigenvalues estimated using the Monte-Carlo data against the expected ones (estimated using the original data) which were used when generating the simulated data; independent 14-component models (Test B); the two dashed lines show the*±2.8σ* range.**

We show the Monte-Carlo plots for the Test A in Figures 7, 8 and 9, while the Figures 10, 11 and 12 show those for the Test B. The results for the Test A on 2D data sets (Figures 7 and 8) indicate very little difference for the low parameter fly wing data, and a more obvious systematic underestimate of covariance (as expected) for the 6 dimensional mouse mandible data (prior to correction).

Further, the results for the Test B (Figures 10 and 11) indicate that even for the mouse mandible data, the values of covariance are significantly different, due to the amplification of initial estimation bias during the process of iterative linear model estimation. The correction process now removes these instabilities bringing estimated covariances back close to the expected sampling limits and symmetrically around the expected correlation line.

Turning to the 3D MS data, for the Test A (Figure 9) the eigenvalues fall inside the allowable range (dashed lines). However for the Test B (Figure 12), the eigenvalues appear to fall under the lower bound. The under-estimation seen is in accordance with a correction factor based upon the number of samples and model complexity (*K*−*J*)/*K*. Unlike the earlier biases this under-estimation does not destabilise the analysis, as a common multiplicative change on all variance estimates leaves the estimated model parameters unaffected.

Note that the equivalent residual distributions estimated here from the conventional Procrustes analysis have no associated correction process and (along with uncorrected estimates from our own algorithm) are probably indicative of anything which could be attempted based upon estimating sample covariances for existing weighted methods.

**Figure 13:**
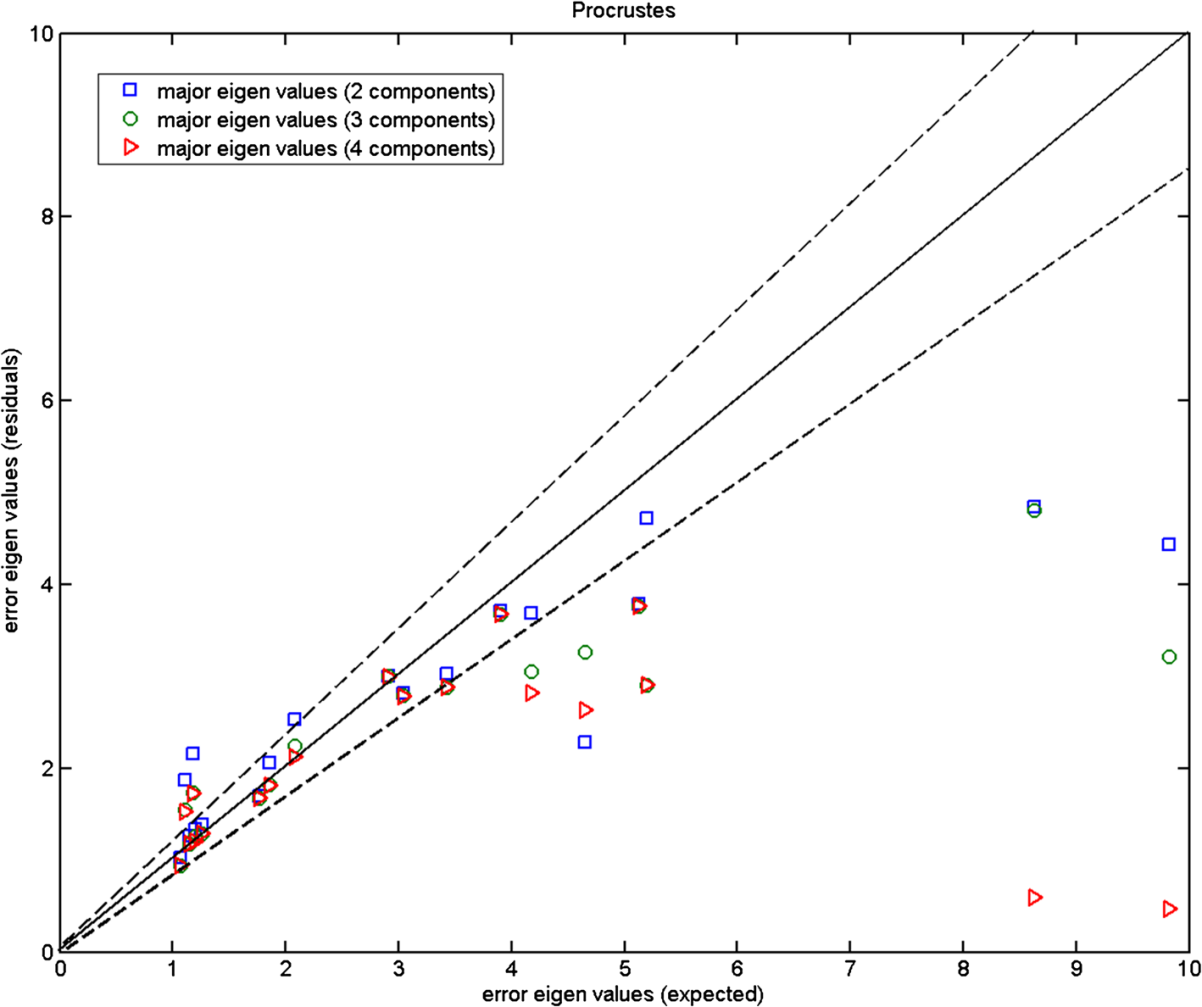
**Fly wing data (P-FL1): error eigenvalues computed using the residuals after Procrustes alignment on the Monte-Carlo data, against the expected ones which were used when generating the simulated data; for 2, 3 and 4 model components; the two dashed lines show the*±2.8σ* range.**

**Figure 14:**
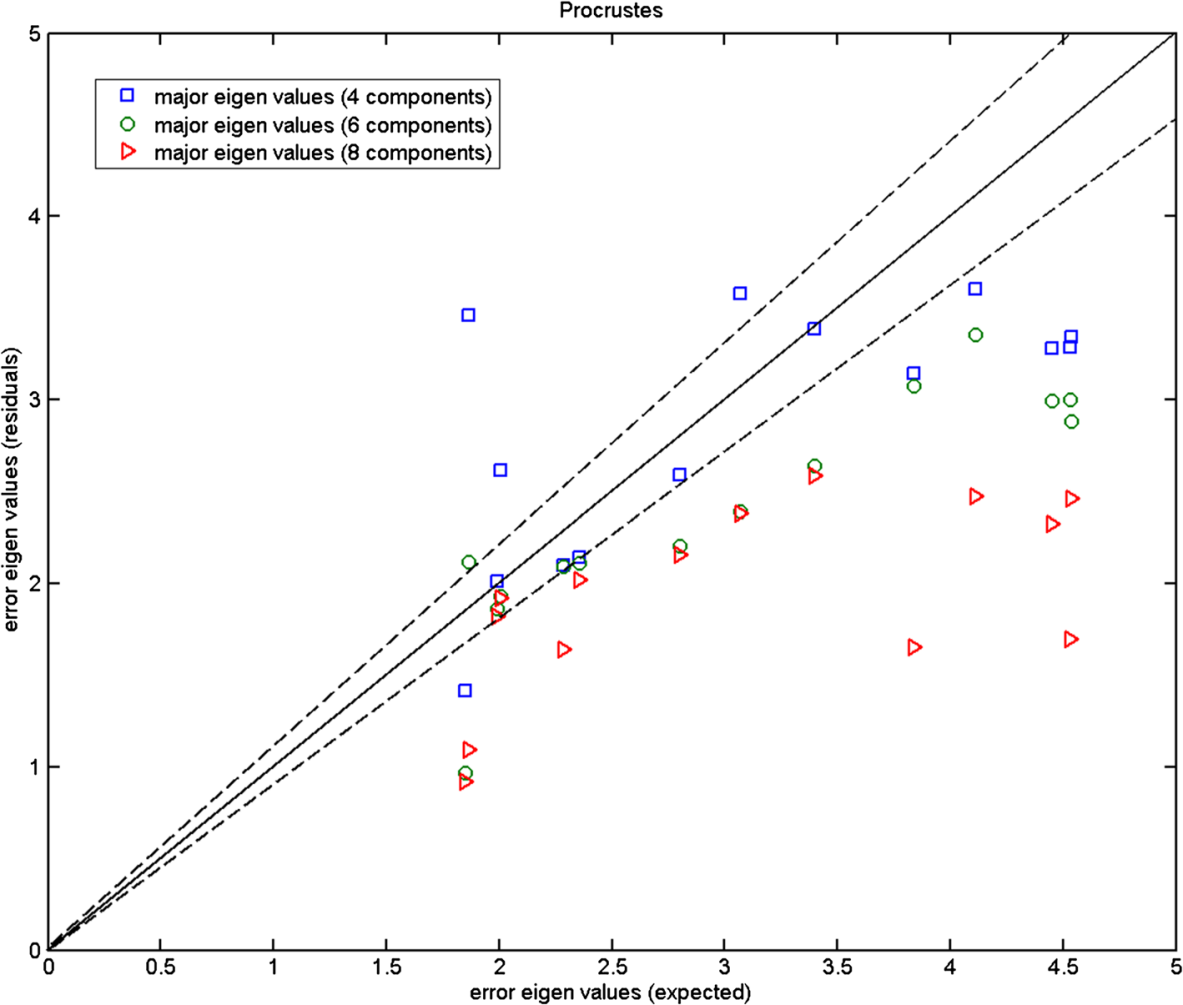
**Mouse mandible data (MM1): error eigenvalues computed using the residuals after Procrustes alignment on the Monte-Carlo data, against the expected ones which were used when generating the simulated data; for 4, 6 and 8 model components; the two dashed lines show the*±2.8σ* range.**

**Figure 15:**
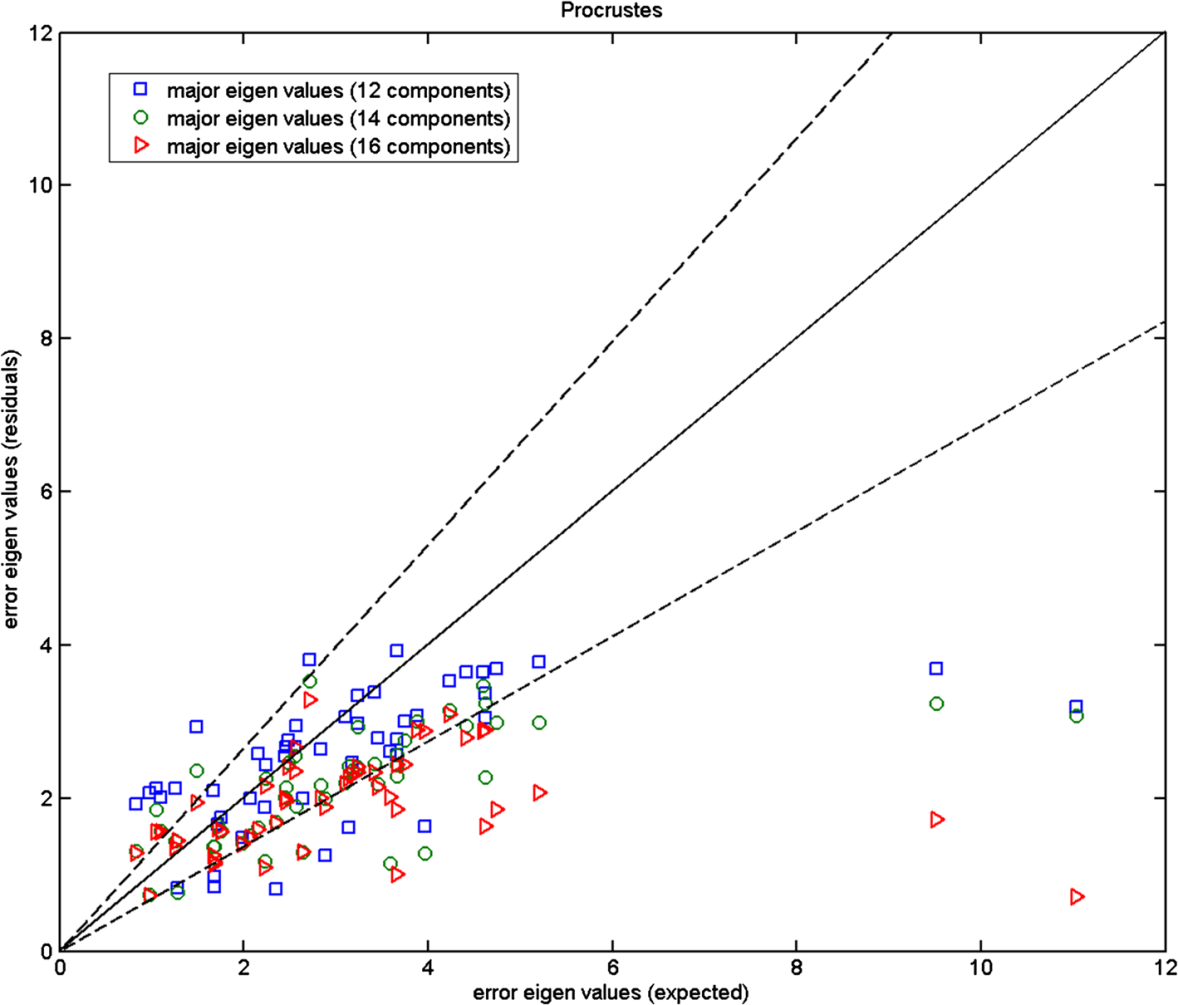
**Mouse skull data (MS): error eigenvalues computed using the residuals after Procrustes alignment on the Monte-Carlo data, against the expected ones which were used when generating the simulated data; for 12, 14 and 16 model components; the two dashed lines show the*±2.8σ* range.**

Here, we compare the results obtained using Procrustes (Figures 13, 14 and 15) to those shown earlier (Figures 10, 11 and 12) using our likelihood-based method. To produce such quantitative results, we apply Procrustes to the same Monte-Carlo data which were generated based on our corrected covariances. Following Procrustes alignment, eigenvalues are computed using the remaining error residuals. These eigenvalues are then plotted against the expected ones, where values on horizontal axis are identical to those used in Figures 10–12. Clearly, (and in contrast to Figures 10–12) in all plots corresponding to Procrustes the measured values are not within the predicted statistical limits (dashed lines). By inference, the linear model vectors constructed using Procrustes are contaminated by random errors associated with poorly measured landmarks, as expected. When compared to the plots from our weighted method with the correction process switched off (e.g. Figure 13 compared to Figure 10), eigenvalues extracted from Procrustes residuals are further away from the expected values. As seen in the figures, changing the number of degrees of freedom of the model is also not sufficient to correct this issue. We can conclude that Procrustes generates a linear model which is a less efficient description of the true information contained in the data.

### Shape analysis

**Figure 16:**
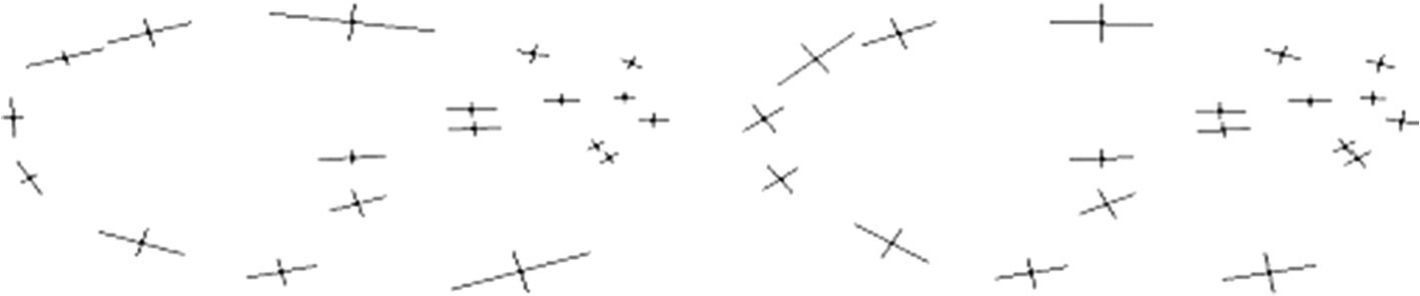
**Fly wing data (P-FL1): error bars (*×20*) estimated using our method (left), and computed from the residuals left using Procrustes (right); 2-component models.**

**Figure 17:**
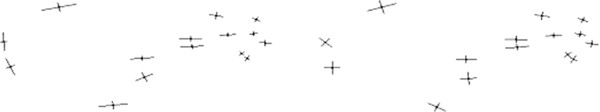
**Fly wing data (FL1): error bars (*×20*) estimated using our method (left), and computed from the residuals left using Procrustes (right); 2-component models.**

**Figure 18:**
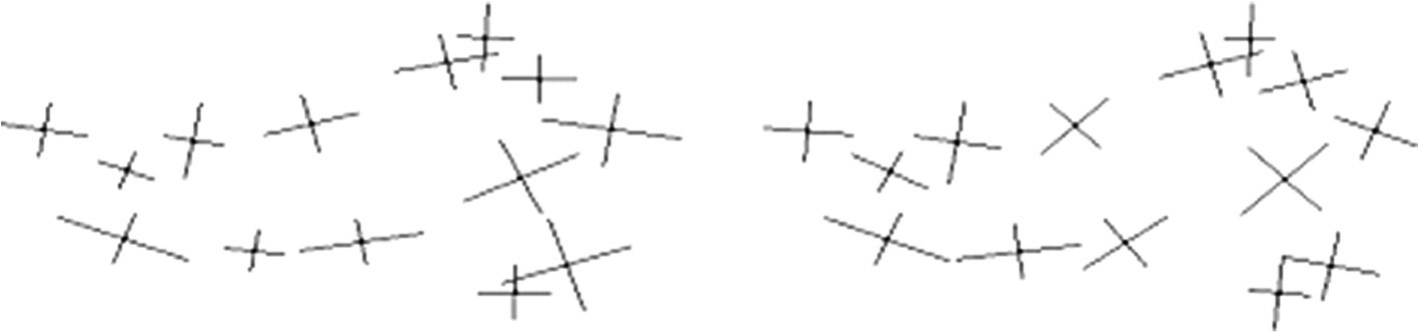
**Mouse mandible data (MM1): error bars (*×20*) estimated using our method (left), and computed from the residuals left using Procrustes (right); 6-component models.**

**Figure 19:**
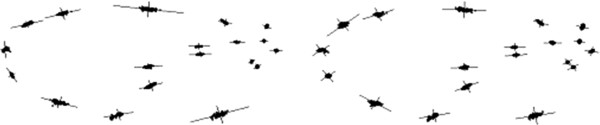
**Fly wing data (P-FL1): error bars (*×20*) estimated using our method (left), and computed from the residuals left using Procrustes (right), with the corresponding aligned data superimposed; 2-component models.**

**Figure 20:**
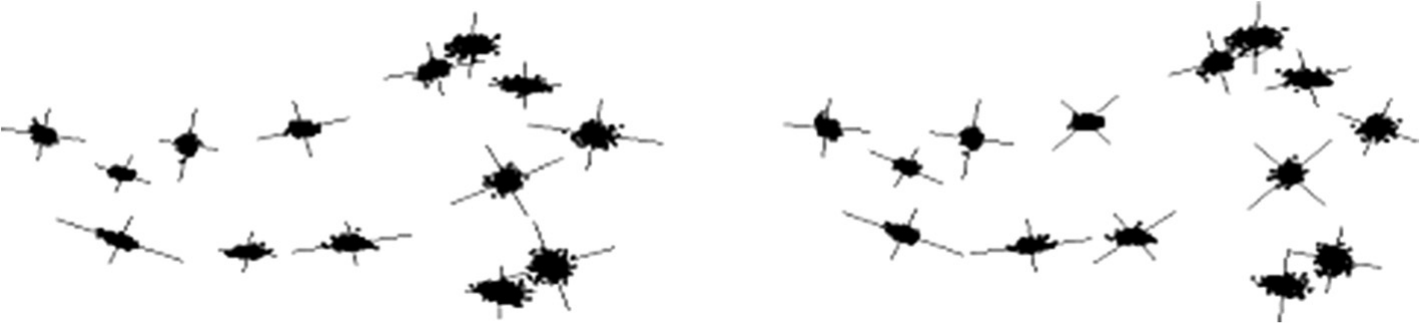
**Mouse mandible data (MM1): error bars (*×20*) estimated using our method (left), and computed from the residuals left using Procrustes (right), with the corresponding aligned data superimposed; 6-component models.**

In Figures 16, 17 and 18, we show the anisotropic error bars computed using the eigenvectors and eigenvalues of the 2×2 covariance matrices for the 2D data sets. All error bars are rescaled for visualisation purposes (see captions). Error bars for each landmark show the extent of an elliptical (non-isotropic) distribution around the corresponding point in the mean shape. Such distributions estimated using our method show exactly why we cannot assume isotropic distributions for the data as assumed in Procrustes. The P-FL1 data used in Figure 16 consists of 19 landmarks (15 + 4) while the FL1 data used in Figure 17 consists of 15 common landmarks only. Using these plots, one can see that the semi-landmarks have anisotropic covariances which match the expected localisation stability. Also we can see how after adding the 4 semi-landmarks the anisotropic errors estimated using our method remain stable, while with Procrustes some change both in orientation and in size, e.g. landmark points 11 and 15 (see Figure 2 for landmark numbers). These error bars are shown again in Figures 19 and 20 with the corresponding aligned data superimposed. In these figures, the extent of error distributions illustrated by the error bars are not expected to match to those illustrated by the alignment, as general biological shape variation and measurement error are independent processes. Although, localisation is determined by local shape characteristics and measurement accuracy plays a role in the overall distribution of landmarks around the mean shape. As a consequence poorly measured landmarks may have a variation about the mean that is dominated by noise.

**Figure 21:**
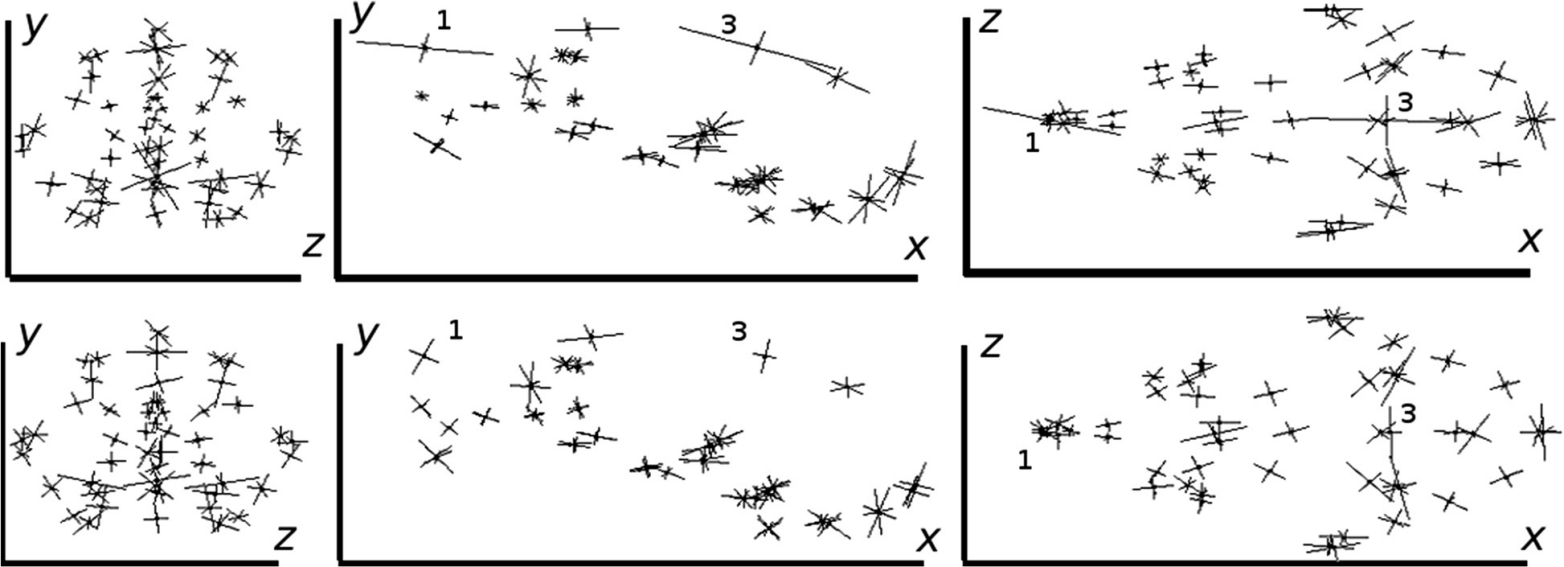
**Mouse skull data: error bars (*×30*) estimated using our covariance-based method (top); and those computed using Procrustes residuals (bottom); 14-component models; projection planes: zy (left), xy (middle) and xz (right).**

**Figure 22:**
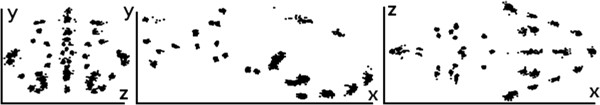
Mouse skull data: aligned data obtained using a 14-component model based on our covariance-based method; projection planes: zy (left), xy (middle) and xz (right).

Turning to 3D data, in Figure 21, we have shown the anisotropic error bars estimated using our method (sub-figures on the top row) and those computed using Procrustes residuals (sub-figures on the bottom row). In Figure 22, we have shown the corresponding aligned data using our method only, as these dense aligned data are visually quite similar for the two methods. In order to display the 3D results we have used their projections on three 2D planes in the original coordinate system.

In both Figures 21 and 22, from the left to the right, we show the projected results on zy, xy and xz planes respectively. Using the mouse skull volume shown in Figure 3, one can see how these viewing planes (zy, xy and xz) correspond to the coronal, sagittal and transverse planes respectively. In these 42 data sets, five had a marked asymmetry of the nasal bones (affecting landmark 1), three had a partially open frontal suture (affecting landmark 3), and one exhibited both of these effects. In Figure 21, one can observe that the largest error bars estimated using our method are for the landmarks 3 and 1. This is consistent with the data clouds corresponding to these landmarks in Figure 22 where in each case some points stand away from the main cloud due to the deformations mentioned above. This is not the case for Procrustes where the error residuals left after alignment for landmarks 3 and 1 show severe underestimation. This is due to the fact that Procrustes translates strong shifts in one landmark position into smaller shifts in all landmarks. However, in this example the observed variation is largely restricted to deformations of the nasal bones (landmark 1) and partially open frontal suture (landmark 3) without displaying noticeable shape changes in other parts of the skull. Hence the larger error bars of our method give in this case a more accurate representation of the observed biological variation. This is in agreement with the results shown earlier in Figure 15 where for two landmarks Procrustes residuals are much smaller than the expected error values (standard deviations) with which the Monte-Carlo data were generated. For our method, however, estimated errors are all comparable to the expected ones as shown earlier in Figure 12. In order to compare the magnitude of errors estimated using our method to those suggested by the repeat data, one should revisit Figure 6. The figure again suggests comparable error estimations. Finally, it is clear from the zy and xz projection planes that expected symmetry is achieved to a large extent in orientation and size for most corresponding error bars (in either method).

**Figure 23:**
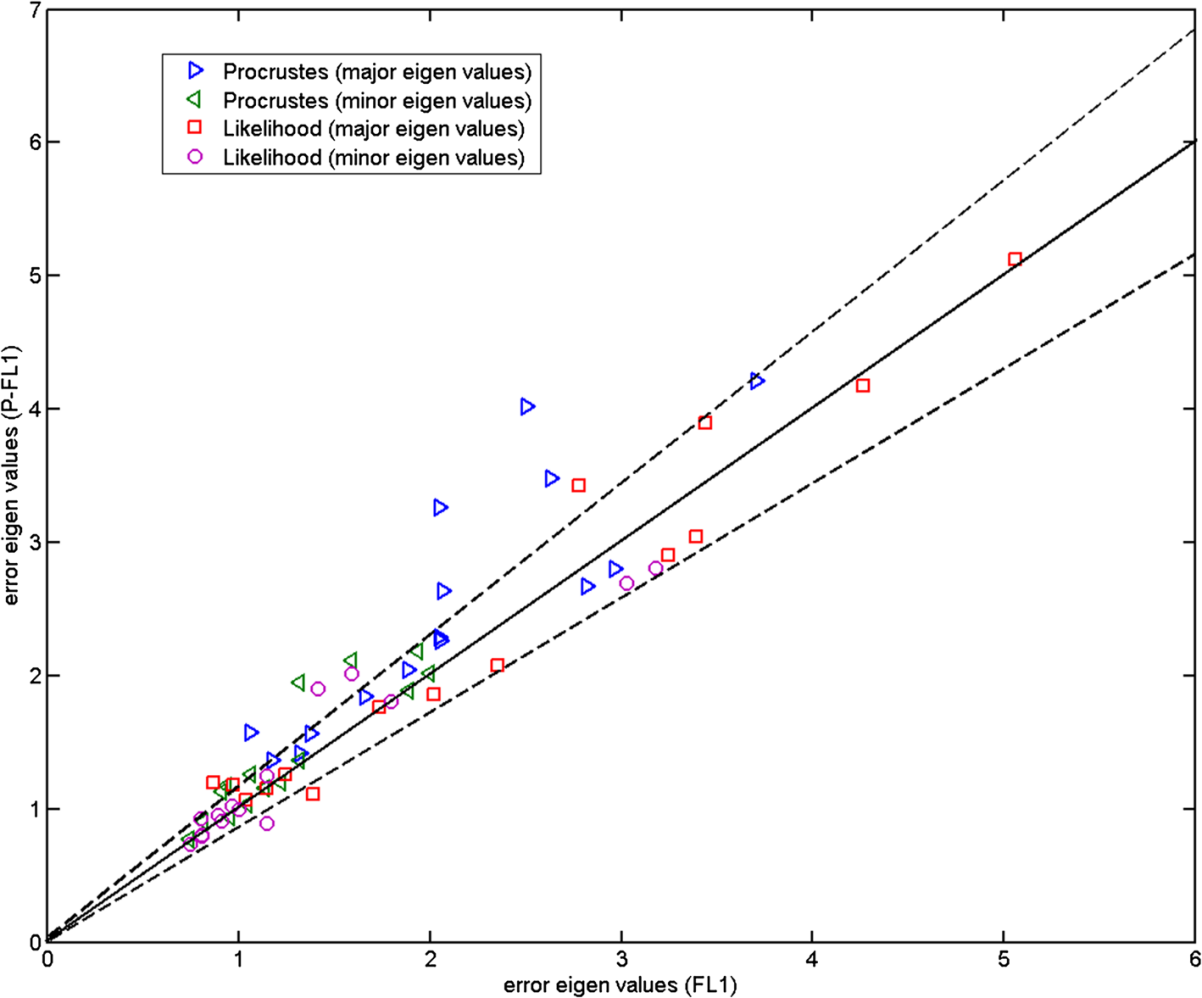
**Fly wing data: error eigenvalues estimated using the likelihood method and those computed using the residuals after Procrustes alignment, when each method is applied to P-FL1 (4 semi-landmarks (16-19) added to FL1) against those when applied to FL1; the plot is for the 15 common landmarks (1-15); the Procrustes results have many points which are well beyond the expected limits, suggesting that model parameters are not consistently determined upon inclusion of semi-landmarks; the two dashed lines show the*±2.8σ* range.**

Now we turn to further comparing our method to Procrustes in a quantitative manner. The inconsistency observed earlier in error bars corresponding to the residuals left after applying Procrustes to the fly wing data (Figures 16 and 17) is displayed more clearly using a scatter plot in Figure 23. Here we plot the eigenvalues corresponding to the 15 common landmarks after the 4 semi-landmarks are added against those without any additional landmarks. We plot this for both the likelihood and Procrustes methods. It is clear here that there are departures from the permitted scatter region when Procrustes is used. This indicates a significant change in the unexplained variance following linear model construction, which itself implies differences in the linear model itself, i.e. the Procrustes model is unstable following the addition of poorly measured landmarks.

**Figure 24:**
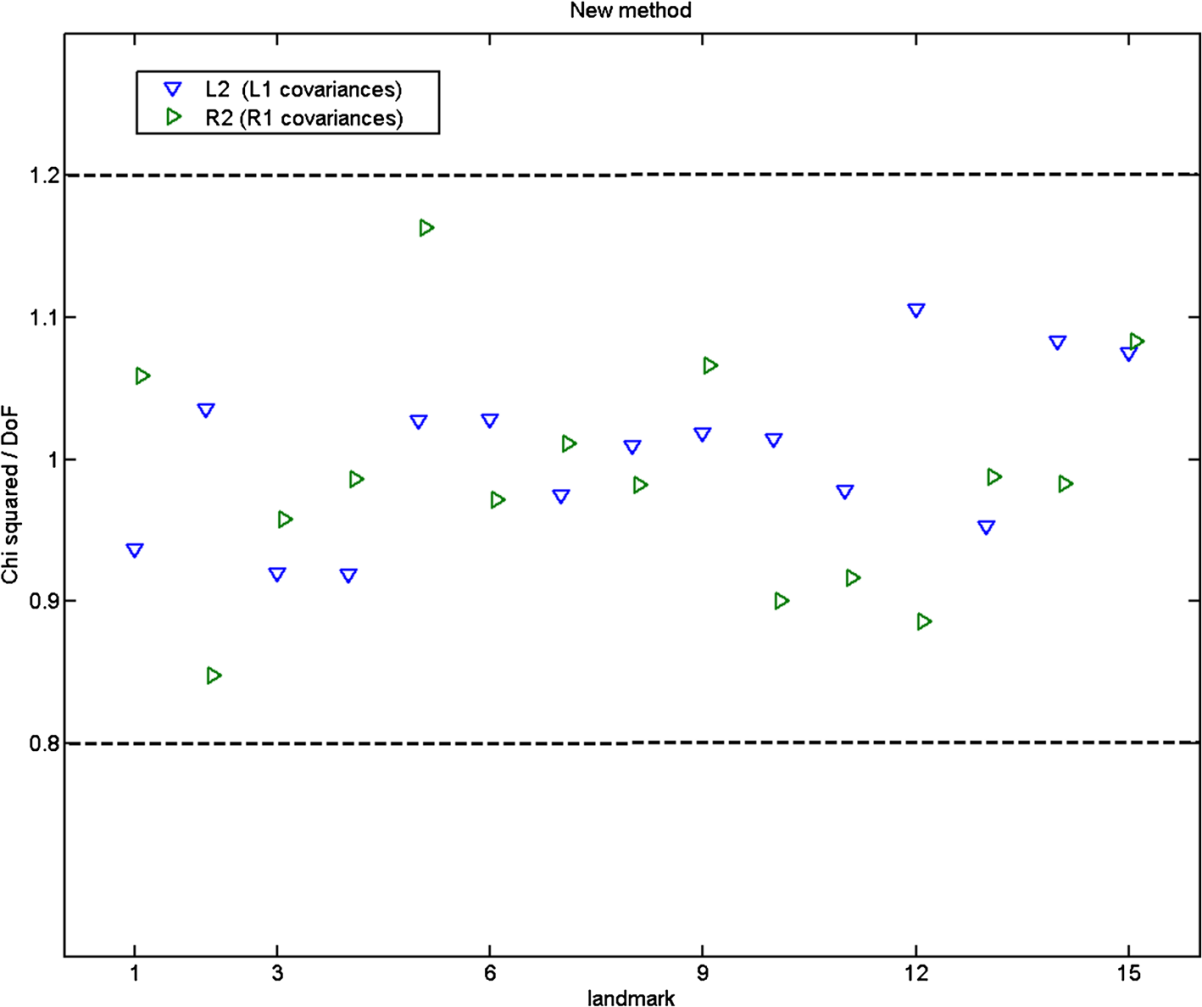
Fly wing data (15 original landmarks): the ***χ***^***2***^***/DoF*** ratios when our method is applied to two sets of repeat fly wing data, FL2 and FR2, using a 3-component model and fixed covariances (Figure 16: top-left) estimated earlier from the FL1 and FR1 data sets respectively; the two dashed lines show the ***±2.8σ*** range.**Fly wing data (15 original landmarks): the*χ***^***2***^***/DoF* ratios when our method is applied to two sets of repeat fly wing data, FL2 and FR2, using a 3-component model and fixed covariances (Figure**16**: top-left) estimated earlier from the FL1 and FR1 data sets respectively; the two dashed lines show the*±2.8σ* range.**

Further, we performed a *χ*^2^ test based upon the construction of corrected covariances on one data set (FL1/FR1) and then used for the calculation of *χ*^2^ for a second data set (FL2/FR2). The corresponding plot for *χ*^2^ test in Figure 24 confirms the stability of our method, as all *χ*^2^/*DoF* values fall in the range expected. Further *χ*^2^ tests (not shown here) with different numbers of data samples and combinations of data sets indicate the appropriateness of the assumed linear model for the fixed number of components.

In Table 2, we list the Fisher Information (FI) value for the two methods and the three data sets studied. Again, for Procrustes, variances used to compute the FI value are obtained from the residuals left after alignment between the data and the simulated linear model. Our method, which is based on likelihood and measurement covariance, gives FI values roughly between two and four times those obtained using Procrustes. The largest difference corresponds to the 3D MS data with 14 model components. As FI is proportional to the quantity of data, this demonstrates that the changes away from the isotropic assumption inherent to Procrustes/PCA has a significant effect on the efficacy of the model, equivalent to having defined only a third as many landmarks from the outset. We can also see in this table the effect of adding 4 semi-landmarks to the 15 original landmarks. The numbers in the parentheses show the contributions from the 4 added landmarks to the total FI values. The reason for the decrease in the FI values after adding 4 landmarks is that we are using the same number of degrees of freedom (DoF) to describe correlations between more points. Also these values are computed for uncorrected covariance estimates, because correction is not available when using Procrustes.

**Table 2:** Fisher information (FI) values: listed for the Procrustes and our method when applied to the fly wing data (P-FL1), mouse mandible data (MM1) and 3D mouse skull data (MS)

**FI value**	**Procrustes**	**Likelihood**
3D Mouse skull data (14-component model)	23.62	111.88
Mouse mandible data (6-component model)	6.60	19.55
Fly wing data (2-component model) 15 points	17.36	29.68
Fly wing data (2-component model) 15 (+4) points	13.81 (+0.88)	25.46 (+2.47)

 Finally, the PCA analysis shows that in fly wing data 3 components can account for about 65% of variance, while for mouse mandibles 6 components are needed to achieve the same level. In both cases, the model order preferred by our analysis is significantly less than the heuristic limit of 90% used by some researchers.

## Conclusions

Our analysis approach has been driven by the requirements of statistical estimation, quantitation and self consistency, i.e. distributions assumed during likelihood construction match the data and estimated parameters match those generating the data. From a more philosophical standpoint we can consider what we are doing when we identify landmark locations and attempt to compare them between sets (shapes). We do not expect that biology manipulates the locations of our chosen landmarks directly, they simply appear to move around as the net effect of distributed developmental and evolutionary influences. Recent considerations of biology have introduced the phrase “palimpsest” [52], as an analogy with repeatedly erasing and rewriting text in an ancient parchment, to describe the way that structures develop. Notice that the initial choice of landmarks is subjective, not only in terms of the features selected but also how we chose to define their locations. A landmark is the result of a localisation procedure (partly influenced by multiple biological considerations) which has an associated positional uncertainty. In this work we have associated the problems of working with semi-landmarks in biological shape analysis as being a consequence of the statistical assumptions implicit to analysis techniques such as Procrustes/PCA. We have implemented a new method which takes appropriate account of measurement and landmark localisation stability in order to obtain a new form of analysis which is consistent with a likelihood-based definition of the alignment and model building tasks. This method can be equivalently interpreted as a redefinition of the landmark location as ghost points.

The conventional interpretation of Procrustes is that the resulting linear model is a pure shape description which can be directly associated with biological processes. Some may argue that extending the approach to weight data, even to accommodate semi-landmarks, breaks with this tradition. However, it is our belief that any distinction between the original landmark and our definition of a ghost point, as locations which are somehow true measurements of biology in one case but not the other, is arbitrary. Re-weighting of data using a covariance is statistically equivalent to modifying the information available by changing the specified set of landmarks. Use of a least-squares measure (which assumes isotropic errors) does not introduce some absolute measurement of biology. Both approaches need to be calibrated using known samples with identifiable biological cause in order to make any scientific interpretation.

Now that we have a specific definition for how to weight landmark data, we can see that using ghost points does not invalidate use of Kendall’s statistics as suggested in [22]. The use of these approaches follows due to scale normalisation of the shape data, it is not an intrinsic property of the use of the original landmarks co-ordinates per-se. We can also re-project scaled (whitened) shapes onto the tangent space defined in the transformed ghost space if we wish, in order to remove local curvature arising from scale normalisation.

Far from there being no objective way to define these covariances [22,24], there are at least three; a) one can estimate them directly from repeatability of measurements (e.g. see [48]); b) they can be directly estimated via conventional statistical means when using likelihood-based landmark location (CRB) (e.g. see [25,26,28]); c) they can be estimated as the unexplained stochastic variation (residuals) in fitted data (as in this paper and e.g. [27]). For the latter, when estimated using residuals of the fitted shape model, we will see contributions additional to the measurement process; this is the stochastic (therefore unmodellable) behaviour of the biology itself. Our results indicate that measurement covariances can be reliably estimated in our data for sample sizes at least as small as 40.

Our result indicate that the new method summarises the information content of the measured data better (improved FI scores), and with more stability than Procrustes/PCA (consistent models are generated following the addition of new points). Although we have not provided empirical evidence in this paper, the expected theoretical advantages of this approach are several; a) as all landmarks of fixed local structure have an associated measurement covariance, the approach described provides a consistent way of incorporating qualitatively different forms of landmark (type I, type II, semi-landmarks, geometric landmarks, etc.) into the analysis; b) provided that landmark stability is well described by a Gaussian distribution, our method removes the instabilities inherent in the analysis due to poorly determined points; c) as the parameters for the linear model are now self-consistently estimated for an identifiable generative scheme (embodied here via Monte-Carlo simulation) it affords the application of an eigenvector analysis statistical rigour; d) it offers the possibility of interpreting the linear modelling process as a statistical approximation, with consequent interpretations of the requirement for the number of linear model components; e) finally, generalisation of the approach would seem to be possible which would support the analysis of dense landmarks on surfaces and curves.

We have also demonstrated how linear model order selection can be performed by comparing baseline reproducibility errors with those estimated from the model. Finally, we have shown how the use of repeated analysis on matched samples can be used to confirm the stability of the estimated anisotropic error. We believe that these tools are sufficient to allow use of this technique in biological studies. More study is needed in order to develop an understanding of the value of our new technique in a greater range of biological analyses.

The methods described in this paper are freely available from the TINA web site [51] via the Geometric Morphometric toolkit, as a system for quality assessment and validation of output data.

## Endnote

^a^Bookstein [53]: “Wherever there is partial registration the true value of a (vector deformation) is inaccessible.”

## Appendix A

### Rotation matrix

Based on the geometry shown in Figure 1, we first calculate the vectors ${\hat{v}}_{a}$, ${\hat{v}}_{b}$ and ${\hat{v}}_{c}$.

$$\begin{array}{lll} {\hat{v}}_{a} & =\frac{P_{2}-P_{1}}{∥P_{2}-P_{1}∥},\;\;\;v_{b}=(P_{3}-P_{1})-[(P_{3}-P_{1}).{\hat{v}}_{a}]{\hat{v}}_{a},\; & \;\\ {\hat{v}}_{c} & ={\hat{v}}_{a}\times \frac{v_{b}}{∥v_{b}∥}\; & \; \end{array}$$

The rotation matrix *R*^*T*^ is hence given by:

$$R^{T}=\left(\begin{array}{lll} {\hat{v}}_{\mathrm{ax}} & {\hat{v}}_{\mathrm{ay}} & {\hat{v}}_{\mathrm{az}}\\ {\hat{v}}_{\mathrm{bx}} & {\hat{v}}_{\mathrm{by}} & {\hat{v}}_{\mathrm{bz}}\\ {\hat{v}}_{\mathrm{cx}} & {\hat{v}}_{\mathrm{cy}} & {\hat{v}}_{\mathrm{cz}} \end{array}\right)$$

### Roll, pitch and aw angles

The multiplication of the rotation matrices *R*_*x*_(*γ*), *R*_*y*_(*β*) and *R*_*z*_(*α*) gives

[hpb!]

$$\;\;R_{\mathrm{xyz}}=\left(\begin{array}{lll} \;\cos\beta \cos\alpha & \;-\cos\beta \sin\alpha & \;\sin\beta \\ \cos\gamma \sin\alpha +\sin\gamma \sin\beta \cos\alpha \; & \;\cos\gamma \cos\alpha -\sin\gamma \sin\beta \sin\alpha \; & \;-\sin\gamma \cos\beta \\ \sin\gamma \sin\alpha -\cos\gamma \sin\beta \cos\alpha \; & \;\sin\gamma \cos\alpha +\cos\gamma \sin\beta \sin\alpha \; & \;\cos\gamma \cos\beta \end{array},\;\right)$$

Hence by enforcing *R*^*T*^=*R*_*xyz*_, it is straightforward to find the rotation angles *α*, *β* and *γ*.

### Direction vectors

At each landmark point *n* with *m*_*nx*_, *m*_*ny*_ and *m*_*nz*_ as the mean coordinates, the rotated vector by angle *α* around the z axis is

$$m^{\prime }=\left(\begin{array}{l} (\cos\alpha )m_{\mathrm{nx}}-(\sin\alpha )m_{\mathrm{ny}}\\ (\sin\alpha )m_{\mathrm{nx}}+(\cos\alpha )m_{\mathrm{ny}}\\ m_{\mathrm{nz}} \end{array}\right)$$

The first derivatives of this vector with respect to *α* gives

$$u_{\alpha }=\left(\begin{array}{l} -(\sin\alpha )m_{\mathrm{nx}}-(\cos\alpha )m_{\mathrm{ny}}\\ (\cos\alpha )m_{\mathrm{nx}}-(\sin\alpha )m_{\mathrm{ny}}\\ 0 \end{array}\right)$$

## Competing interests

The authors declare that they have no competing interests.

## Authors’ contributions

HR undertook software and methods development, performed experiments, and produced the final version of the manuscript and responses to reviews. NAT conceived the new statistical methods for shape analysis and provided technical project coordination. PAB developed the automatic landmarking software and provided maintenance of software libraries, web pages and infrastructure. DT provided overall scientific management and coordination of the project. ACS participated in acquisition of datasets including manual/automatic landmark identification. All authors read and approved the final manuscript.
